# Synthesis and Biological Evaluation of Novel Triple-Modified Colchicine Derivatives as Potent Tubulin-Targeting Anticancer Agents

**DOI:** 10.3390/cells7110216

**Published:** 2018-11-19

**Authors:** Urszula Majcher, Greta Klejborowska, Magdalena Kaik, Ewa Maj, Joanna Wietrzyk, Mahshad Moshari, Jordane Preto, Jack A. Tuszynski, Adam Huczyński

**Affiliations:** 1 Department of Bioorganic Chemistry, Faculty of Chemistry, Adam Mickiewicz University, Umultowska 89b, 61-614 Poznan, Poland; urszula.majcher@amu.edu.pl (U.M.); greta.klejborowska@amu.edu.pl (G.K.); magdakaik@gmail.com (M.K.); 2 Hirszfeld Institute of Immunology and Experimental Therapy, Polish Academy of Sciences, Rudolfa Weigla 12, 53-114 Wrocław, Poland; ewa.maj@iitd.pan.wroc.pl (E.M.); wietrzyk@iitd.pan.wroc.pl (J.W.); 3 Depertment of Chemistry, University of Alberta, Edmonton, AB T6G 1Z2, Canada; moshari@ualberta.ca; 4 Depertment of Oncology, University of Alberta, Edmonton, AB T6G 1Z2, Canada; preto@ualberta.ca (J.P.); jack.tuszynski@gmail.com (J.A.T.); 5 DIMEAS, Politecnico di Torino, Corso Duca degli Abruzzi 24, 10129 Turin, Italy

**Keywords:** colchicine binding site inhibitor, tubulin-targeting agent, antimitotic agent, antiproliferative activity, thiocolchicine, natural compounds

## Abstract

Specific modifications of colchicine followed by synthesis of its analogues have been tested in vitro with the objective of lowering colchicine toxicity. Our previous studies have clearly shown the anticancer potential of double-modified colchicine derivatives in C-7 and C-10 positions. Here, a series of novel triple-modified colchicine derivatives is reported. They have been obtained following a four-step strategy. In vitro cytotoxicity of these compounds has been evaluated against four human tumor cell lines (A549, MCF-7, LoVo, and LoVo/DX). Additionally, the mode of binding of the synthesized compounds was evaluated in silico using molecular docking to a 3D structure of β-tubulin based on crystallographic data from the Protein Data Bank and homology methodology. Binding free energy estimates, binding poses, and MlogP values of the compounds were obtained. All triple-modified colchicine derivatives were shown to be active at nanomolar concentrations against three of the investigated cancer cell lines (A549, MCF-7, LoVo). Four of them also showed higher potency against tumor cells over normal cells as confirmed by their high selectivity index values. A vast majority of the synthesized derivatives exhibited several times higher cytotoxicity than colchicine, doxorubicin, and cisplatin.

## 1. Introduction

**Figure 1:**
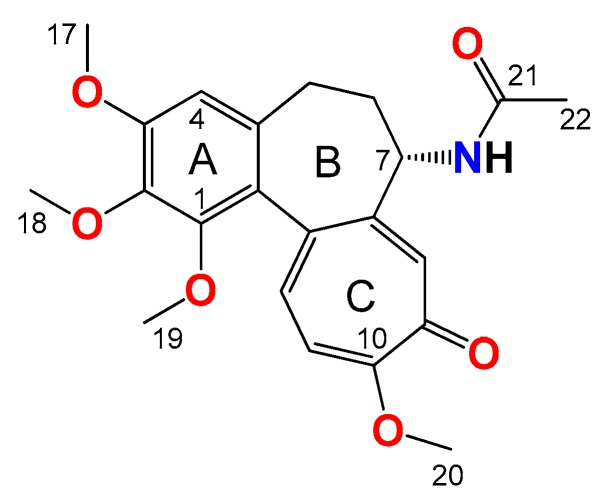
Structure of colchicine (**1**).

Colchicine (**1**, Figure 1) is one of the oldest therapeutic substances known to mankind. Although its medical properties have been known for centuries, the drug was approved for clinical use by the United States Food and Drug Administration only in 2009 [1]. Clinically, colchicine is approved and used for the treatment of familial Mediterranean fever, Behcet’s disease, acute gout, chondrocalcinosis, and other types of microcrystalline arthritis [2,3,4,5,6]. Other therapeutic indications include primary biliary cirrhosis, psoriasis, amyloidosis, various forms of dermatitis, relapsing polychondritis, necrotizing vasculitis, Sweet’s syndrome, leukocytoclastic vasculitis, and cardiovascular diseases, such as particular pericarditis, atrial fibrillation caused by inflammation, and ischemic episodes [7,8,9,10,11,12]. Colchicine is of particular interest in a broader context, especially as a cancer chemotherapy agent, due to its antimitotic properties. It has played an important role in studies of mitosis and the therapeutic potential of the colchicine binding site has been investigated for chemotherapy applications. However, its clinical use is often hampered by relatively high general toxicity, which results from its accumulation in the gastrointestinal tract, as well as neurotoxicity [13,14,15].

Many efforts have been made to develop colchicine analogs with the goal of reducing toxicity and increasing bioavailability. Additionally, diverse structure-activity relationship studies have been performed to elucidate the structural features required for tubulin binding. Substitution of methoxyl group in the C-10 position by a thiomethyl group increases molecular stability and thiocolchicine binds to tubulin more rapidly than colchicine itself [16,17,18,19,20,21]. In 2011 Hiromitsu Takayama’s research group [22] published results of their studies on C-4 halogen substituted colchicine derivatives. Some of them exhibited more potent cytotoxicity for tumor cells compared to **1**. In our previous studies, we prepared double-modified, in the C-7 and C-10 positions, colchicine derivatives and evaluated their cytotoxicity [23]. Encouraged by these results, we decided to further develop the concept of diversified urethane substituents in the C-7 position and combine it with bromination in the C-4 position. We synthesized a series of triple-modified 4-bromo-*N*-deacetyl-7-carbamatethiocolchicines maintaining the stereochemistry of the C-7 center, which is critical for antimitotic activity [15,21].

## 2. Materials and Methods

### 2.1. General

All precursors for the synthesis and solvents were obtained from Sigma-Aldrich (Merck KGaA, Saint Louis, MO, USA) and were used as received without further purification. Spectral grade solvent (CDCl_3_) was stored over 3 Å molecular sieves for several days. Thin layer chromatography (TLC) was carried out on precoated plates (TLC silica gel 60 F254, Aluminum Plates Merck, Merck KGaA, Saint Louis, MO, USA) and spots were detected by illumination with a UV lamp. All the solvents used in flash chromatography were of HPLC grade (CHROMASOLV from Sigma-Aldrich, Merck KGaA, Saint Louis, MO, USA) and were used as received. The elemental analysis of compounds was carried out on Vario ELIII (Elementar, Langenselbold, Germany).

### 2.2. Spectroscopic Measurements

The ^1^H, ^13^C spectra were recorded on a Varian VNMR-S 400 MHz spectrometer (Varian, Inc., Palo Alto, CA, USA). ^1^H-NMR measurements of **2**–**8** (0.07 mol dm^−3^) in CDCl_3_ were carried out at the operating frequency 402.64 MHz. The error of the chemical shift value was 0.01 ppm. The ^13^C-NMR spectra were recorded at the operating frequency 101.25 MHz. The error of chemical shift value was 0.1 ppm. All spectra were locked to deuterium resonance of CDCl_3_. The ^1^H- and ^13^C-NMR spectra are shown in the Supplementary Materials.

The FT-IR spectra of **2**–**8** in the mid-infrared region were recorded in KBr. The spectra were taken with an IFS 113v FT-IR spectrophotometer (Bruker, Karlsruhe, Germany) equipped with a DTGS detector; resolution 2 cm^–1^, NSS = 64. The Happ–Genzel apodization function was used.

The ESI (electrospray ionisation) mass spectra were recorded also on a Waters/Micromass (Waters Corporation, Manchester, UK) ZQ mass spectrometer equipped with a Harvard Apparatus syringe pump. The samples were prepared in dry acetonitrile (5 × 10^−5^ mol dm^−3^). The sample was infused into the ESI source using a Harvard pump at a flow rate of 20 mL min^−1^. The ESI source potentials were: capillary 3 kV, lens 0.5 kV, and extractor 4 V. The standard ESI mass spectra were recorded at the cone voltages: 10 and 30 V. The source temperature was 120 °C and the desolvation temperature was 300 °C. Nitrogen was used as the nebulizing and desolvation gas at flow-rates of 100 dm^3^ h^−1^. Mass spectra were acquired in the positive ion detection mode with unit mass resolution at a step of 1 *m*/*z* unit. The mass range for ESI experiments was from *m*/*z* = 100 to *m*/*z* = 1000, as well as from *m*/*z* = 200 to *m*/*z* = 1500.

### 2.3. Synthesis

#### 2.3.1. Synthesis of **2**

**Figure 2:**
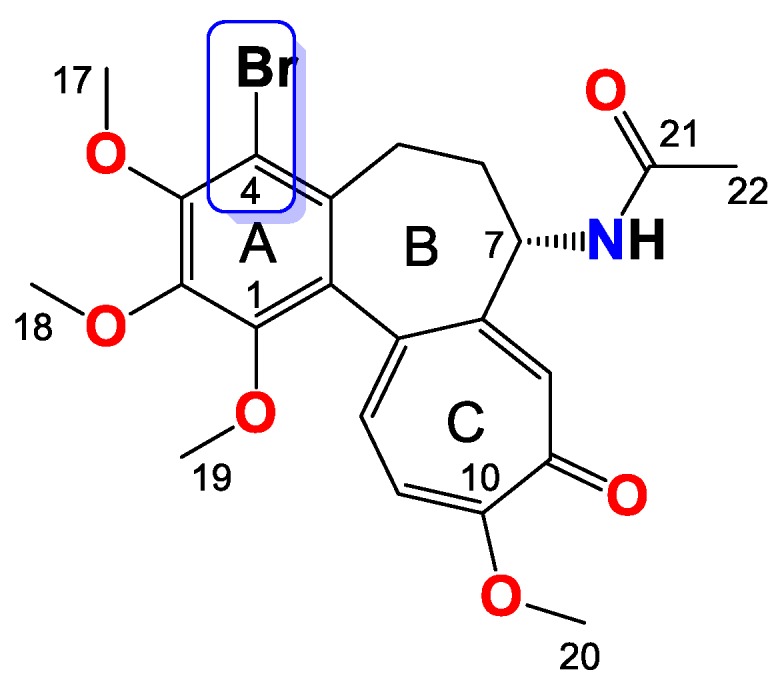
Structure of compound **2**.

A mixture of N-bromosuccinimide (NBS, 279 mg, 1.57 mmol) and **1** (500 mg, 1.25 mmol) in acetonitrile was stirred at RT under nitrogen atmosphere for the 72 h. Reaction time was determined by TLC. The reaction was quenched with saturated aqueous Na_2_S_2_O_3_. The whole mixture was extracted four times with CH_2_Cl_2_, and the combined organic layers were dried over MgSO_4_, filtered, and evaporated under reduced pressure. The residue was purified by CombiFlash^®^ (EtOAc/MeOH, increasing concentration gradient) to give **2** (MW = 478.3 g/mol, Figure 2) as amorphous yellow solid with yield 95% (569 mg) [22]. ^1^H-NMR (403 MHz, CDCl_3_) δ 8.02 (s, 1H), 7.58 (s, 1H), 7.30 (d, *J* = 10.7 Hz, 1H), 6.88 (d, *J* = 11.1 Hz, 1H), 4.59–4.49 (m, 1H), 4.03 (s, 3H), 3.99 (s, 3H), 3.96 (s, 3H), 3.63 (s, 3H), 3.27 (dd, *J* = 13.0, 4.3 Hz, 1H), 2.26 (dd, *J* = 13.1, 5.2 Hz, 1H), 2.18 (d, *J* = 2.4 Hz, 1H), 1.99 (s, 3H), 1.78 (s, 1H) ppm. ^13^C-NMR (101 MHz, CDCl_3_) δ 179.5, 170.2, 164.4, 151.8, 151.1, 150.4, 146.6, 135.8, 135.7, 133.4, 130.2, 130.0, 113.5, 112.4, 61.5, 61.5, 61.0, 56.5, 52.6, 34.5, 28.9, 22.8 ppm. FT-IR (KBr pellet): 3274, 2936, 1662, 1617, 1589, 1565, 1462, 1411, 1398, 1350, 1270, 1250, 1172, 1137, 1080, 1018 cm^−1^. ESI-MS (*m*/*z*): [M + Na]^+^ calcd. 500, found 500, [M + 2 + Na]^+^ calcd. 502, found 502, [2M + 2 + Na]^+^ calcd. 979, found 979, [2M + Na]^+^ calcd. 977, found 977, [2M + 4 + Na]^+^ calcd. 981, found 981.

#### 2.3.2. Synthesis of **3**

**Figure 3:**
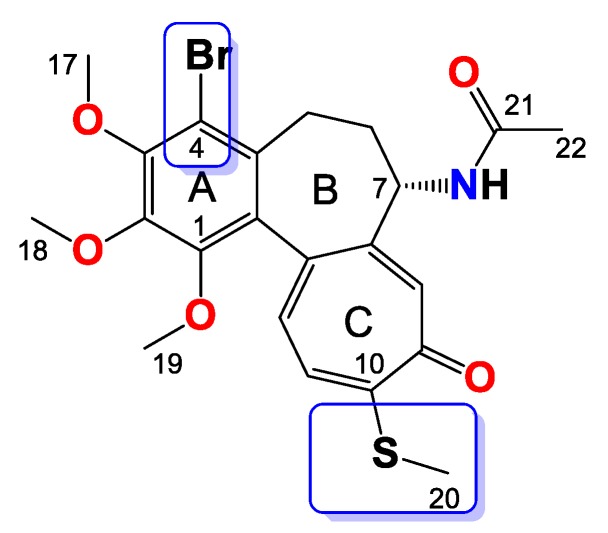
Structure of compound **3**.

To a mixture of **2** (500 mg, 1.01 mmol) in MeOH/water (1/1, *v*/*v*, 5 mL), the sodium methanethiolate (solution 21% in H_2_O, 0.79 mL, 2.1 mmol) was added. The mixture was stirred in at RT for 72 h. Reaction time was determined by TLC. After that time the reaction mixture was quenched by the addition of water (150 mL). The whole mixture was extracted four times with CH_2_Cl_2_, and the combined organic layers were dried over MgSO_4_, filtered, and evaporated under reduced pressure. The residue was purified by CombiFlash^®^ (hexane/EtOAc (1/1), then EtOAc/MeOH, increasing concentration gradient) to give **3** (Figure 3, MW = 494.4 g/mol) as amorphous yellow solid with yield 75% (388 mg) [24].^1^H-NMR (403 MHz, CDCl_3_) δ 7.68 (d, *J* = 6.6 Hz, 1H), 7.42 (s, 1H), 7.26 (d, *J* = 9.6 Hz, 1H), 7.08 (d, *J* = 10.8 Hz, 1H), 4.61–4.52 (m, 1H), 3.99 (s, 3H), 3.97 (s, 3H), 3.63 (s, 3H), 3.27 (d, *J* = 8.0 Hz, 1H), 2.45 (s, 3H), 2.25 (dt, *J* = 13.4, 7.9 Hz, 2H), 2.01 (s, 3H), 1.85 (dd, *J* = 6.7, 4.1 Hz, 1H) ppm. ^13^C-NMR (101 MHz, CDCl_3_) δ 182.4, 170.0, 159.2, 151.2, 151.0, 150.4, 146.6, 137.4, 134.8, 133.4, 130.2, 128.1, 126.3, 113.5, 61.6, 61.5, 61.0, 52.2, 34.5, 29.0, 22.9, 15.2 ppm. FT-IR (KBr pellet): 3267, 2930, 1659, 1603, 1559, 1462, 1410, 1347, 1138, 1074, 1053, 1014 cm^−1^. ESI-MS (*m*/*z*): [M + H]^+^ calcd 494, found 494, [M + 2 + H]^+^ 496, found 496, [M + Na]^+^ calcd 516, found 516, [M + 2 + Na]^+^ calcd 518, found 518, [2M + H]^+^ calcd 989, found 989, [2M + 2 + H]^+^ calcd 991, found 991, [2M + Na]^+^ calcd 1011, found 1011, [2M + 2 + Na]^+^ calcd 1013, found 1013.

#### 2.3.3. Synthesis of **4**

**Figure 4:**
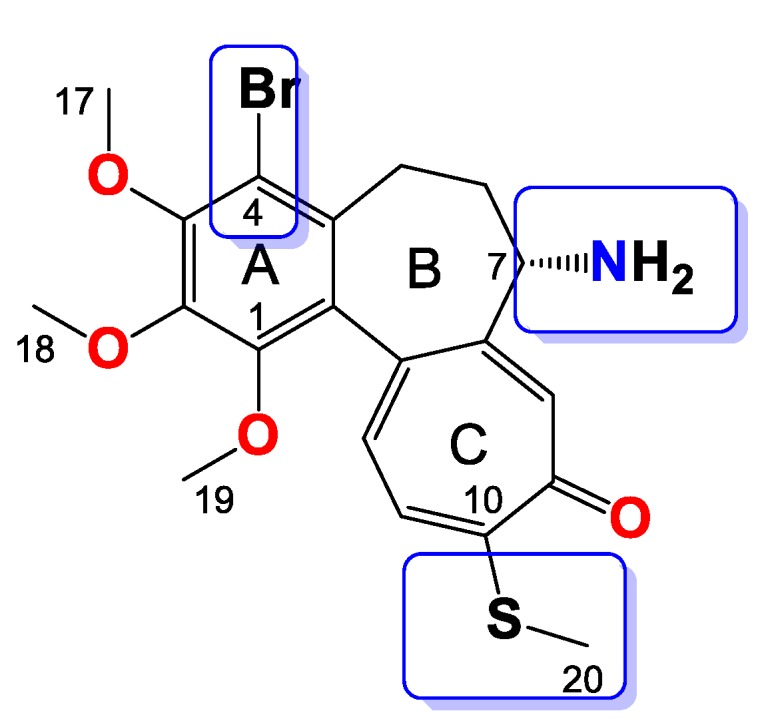
Structure of compound **4**.

Compound **4** (Figure 4) was prepared from **3** by hydrolysis with 2 N HCl. To a solution of compound **3** (500 mg, 1.01 mmol) in MeOH (3 mL), the 2 N HCl solution (5 mL) was added. The mixture was stirred at 90 °C for 72 h. Reaction time was determined by TLC. After that time the reaction mixture was quenched by the addition of water (100 mL). The whole mixture was extracted four times with CH_2_Cl_2_, and the combined organic layers were dried over MgSO_4_, filtered, and evaporated under reduced pressure. The residue was purified by CombiFlash^®^ (EtOAc/MeOH, increasing concentration gradient) to give 4 (MW = 452.4 g/mol) as amorphous brownish solid with yield 80% (366 mg) [25]. ^1^H-NMR (403 MHz, CDCl_3_) δ 7.52 (s, 1H), 7.05 (d, *J* = 10.3 Hz, 1H), 6.94 (d, *J* = 10.7 Hz, 1H), 3.88 (s, 3H), 3.88 (s, 3H), 3.55 (s, 3H), 3.54–3.51 (m, 1H), 3.16–3.10 (m, 1H), 2.37 (s, 3H), 2.24–2.15 (m, 2H), 1.50–1.45 (m, 1H) ppm. ^13^C-NMR (101 MHz, CDCl_3_) 182.5, 158.7, 151.1, 149.9, 146.1, 137.1, 134.3, 134.0, 129.9, 129.2, 125.5, 113.2, 61.3, 61.0, 61.0, 53.4, 38.2, 29.6, 15.1 ppm. FT-IR (KBr pellet): 3378, 3315, 2935, 1605, 1557, 1462, 1409, 1345, 1248, 1196, 1138, 1083, 1016 cm^−1^. ESI-MS (*m*/*z*): [M + Na]^+^ calcd. 474, found 474, [M + 2 + Na]^+^ calcd. 476, found 476.

#### 2.3.4. General Procedure for the Synthesis of Colchicine Derivatives (**5**–**12**)

Compounds **5**–**12** were obtained directly from compound **4**. To a solution of compound **4** (100 mg, 0.22 mmol) in tetrahydrofuran (THF, 5 mL) cooled to the 0 °C temperature, the following compounds were added: Et_3_N (1 mL, 7 mmol), and triphosgene (69 mg, 0.23 mmol). The mixture was first stirred at 0 °C temperature for 20 min and then for the next 20 min at RT. After that time respective alcohol (11 mmol) was added and the mixture was stirred at RT for the next 48 h. Reaction time was determined by TLC. The solution was filtered to remove triethylamine hydrochloride. The THF was evaporated and the residue was quenched by the addition of CH_2_Cl_2_ (100 mL) and was washed sequentially with a solution of HCl(aq) (0.5 M) and then with water. The organic layer was evaporated to dryness under reduced pressure and purified by CombiFlash^®^ (hexane/ethyl acetate, increasing concentration gradient) to give respective compounds as amorphous yellow solids (**5**–**12**).

##### Compound **5**

**Figure 5:**
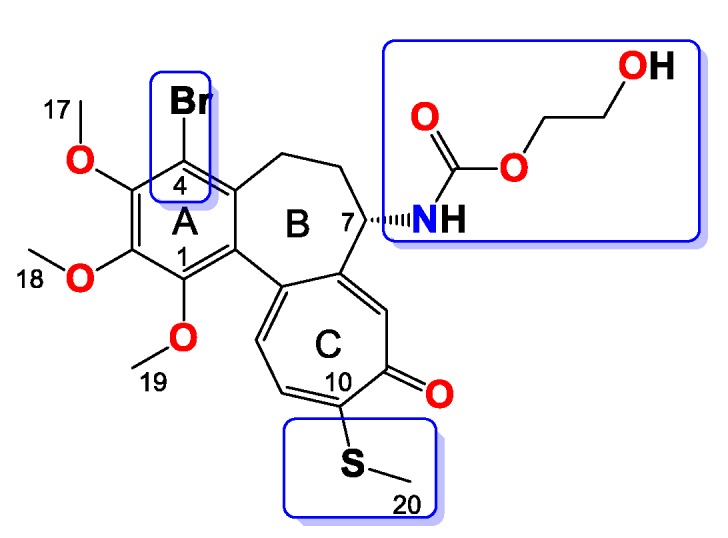
Structure of compound **5**.

Amorphous yellowish brown solid, yield 55 mg, 46%, MW = 539.1 g/mol (Figure 5). ^1^H-NMR (403 MHz, CDCl_3_) δ 7.43 (s, 1H), 7.23 (d, *J* = 10.3 Hz, 1H), 7.07 (d, *J* = 10.5 Hz, 1H), 6.55 (d, *J* = 6.9 Hz, 1H), 4.38–4.27 (m, 1H), 4.05 (dd, *J* = 8.7, 4.1 Hz, 2H), 3.96 (s, 3H), 3.95 (s, 3H), 3.67–3.63 (m, 2H), 3.59 (s, 3H), 3.29–3.22 (m, 1H), 2.45 (s, 3H), 2.33–2.21 (m, 2H), 1.83 (dd, *J* = 10.4, 6.1 Hz, 1H) ppm. ^13^C-NMR (101 MHz, CDCl_3_) δ 182.4, 159.2, 156.0, 151.2, 150.6, 150.3, 146.5, 137.0, 134.7, 133.5, 130.0, 128.5, 126.2, 113.5, 66.9, 61.4, 61.4, 61.1, 61.0, 53.7, 34.9, 29.0, 15.1 ppm. FT-IR (KBr pellet): 3295, 2936, 1719, 1607, 1547, 1463, 1410, 1348, 1324, 1288, 1249, 1154, 1141, 1083, 1062, 1020 cm^−1^. ESI-MS (*m*/*z*): [M + H]^+^ calcd. 540, found 540, [M + 2 + H]^+^ calcd. 542, found 542, [M + Na]^+^ calcd. 562, found 562, [M + 2 + Na]^+^ calcd. 564, found 564.

##### Compound **6**

**Figure 6:**
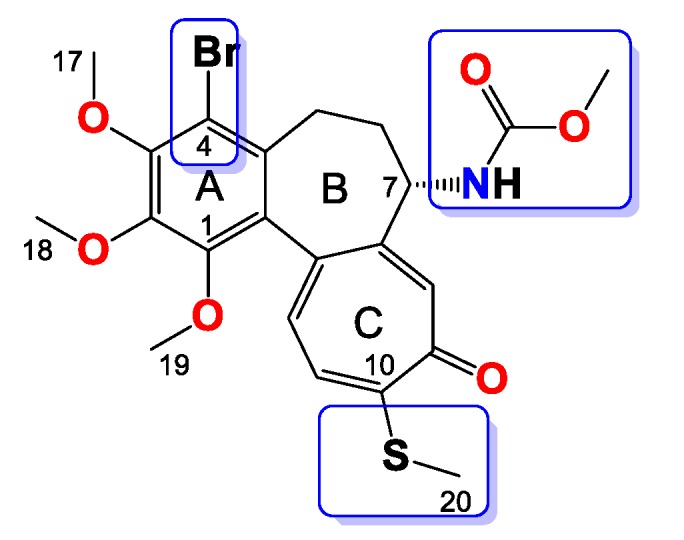
Structure of compound **6**.

Amorphous yellowish brown solid, yield 47 mg, 42%, MW = 509.1 g/mol (Figure 6). ^1^H-NMR (403 MHz, CDCl_3_) δ 7.32 (s, 1H), 7.20 (d, *J* = 10.3 Hz, 1H), 7.03 (d, *J* = 10.4 Hz, 1H), 5.60 (d, *J* = 7.2 Hz, 1H), 4.37–4.27 (m, 1H), 3.97 (s, 3H), 3.95 (s, 3H), 3.60 (s, 3H), 3.59 (s, 3H), 3.25 (d, *J* = 8.0 Hz, 1H), 2.43 (s, 3H), 2.25 (dd, *J* = 6.8, 3.4 Hz, 2H), 1.76–1.66 (m, 1H) ppm. ^13^C-NMR (101 MHz, CDCl_3_) δ 182.3, 159.3, 156.0, 151.2, 150.4, 149.9, 146.6, 136.6, 134.5, 133.4, 130.1, 128.5, 125.8, 113.5, 61.5, 61.4, 61.0, 53.6, 52.3, 35.2, 29.0, 15.2 ppm. FT-IR (KBr pellet): 3297, 2932, 1725, 1608, 1551, 1463, 1410, 1348, 1323, 1289, 1248, 1197, 1153, 1020 cm^−1^. ESI-MS (*m*/*z*): [M + H]^+^ calcd. for 510, found 510, [M + 2 + H]^+^ calcd. 512, found 512, [M + Na]^+^ calcd. 534, found 534, [M + 2 + Na]^+^ calcd. 536, found 536.

##### Compound **7**

**Figure 7:**
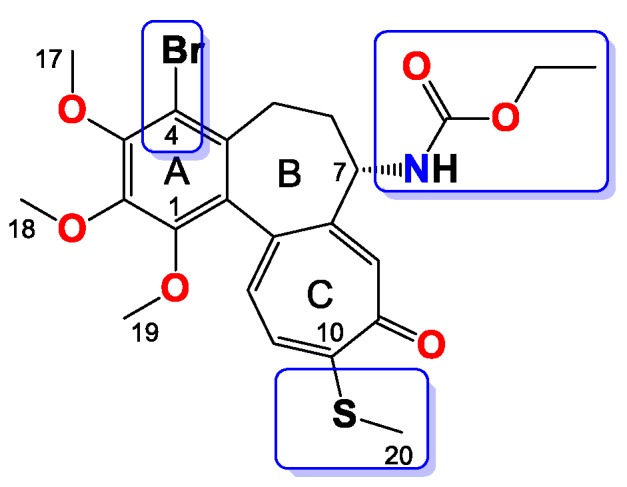
Structure of compound **7**.

Amorphous yellowish brown solid, yield 35 mg, 30%, MW = 524.4 g/mol (Figure 7). ^1^H-NMR (403 MHz, CDCl_3_) δ 7.32 (s, 1H), 7.20 (d, *J* = 10.3 Hz, 1H), 7.03 (d, *J* = 10.5 Hz, 1H), 5.35 (d, *J* = 7.2 Hz, 1H), 4.37–4.28 (m, 1H), 4.02 (dd, *J* = 7.1, 2.2 Hz, 1H), 3.98 (s, 3H), 3.96 (s, 3H), 3.94–3.90 (m, 1H), 3.61 (s, 3H), 3.27 (d, *J* = 8.9 Hz, 1H), 2.44 (s, 3H), 2.30–2.24 (m, 2H), 1.72–1.65 (m, 1H), 1.19 (t, *J* = 7.1 Hz, 3H) ppm. ^13^C-NMR (101 MHz, CDCl_3_) δ 182.3, 159.2, 155.5, 151.1, 150.4, 149.9, 146.6, 136.6, 134.5, 133.4, 130.09, 128.5, 125.8, 113.5, 61.5, 61.4, 61.2, 61.0, 53.4, 35.3, 29.0, 15.2, 14.4 ppm. FT-IR (KBr pellet): 3303, 2935, 1716, 1608, 1550, 1460, 1406, 1346, 1322, 1248, 1150, 1084, 1020 cm^−1^. ESI-MS (*m*/*z*): [M + H]^+^ calcd. for 524, found 524, [M + 2 + H]^+^ calcd. 526, found 526, [M + Na]^+^ calcd. 546, found 546, [M + 2 + Na]^+^ calcd. 548, found 548.

##### Compound **8**

**Figure 8:**
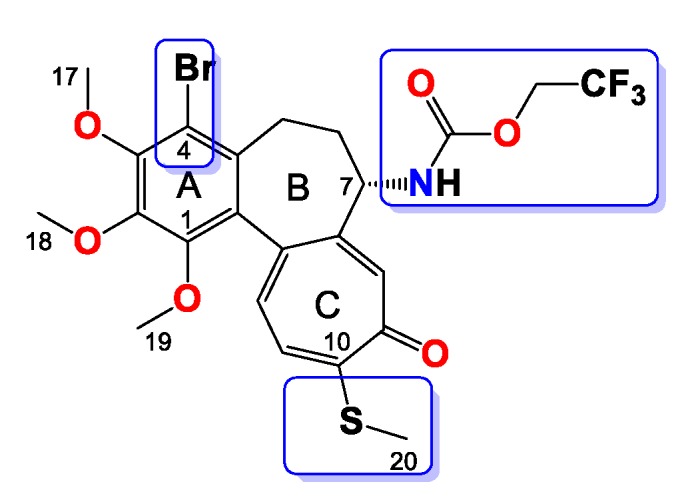
Structure of compound **8**.

Amorphous yellowish solid, yield 49 mg, 38%, MW = 578.4 g/mol (Figure 8). ^1^H-NMR (403 MHz, CDCl_3_) δ 7.37 (s, 1H), 7.22 (d, *J* = 10.3 Hz, 1H), 7.06 (d, *J* = 10.6 Hz, 1H), 6.18 (d, *J* = 7.4 Hz, 1H), 4.48 (dq, *J* = 12.6, 8.5 Hz, 1H), 4.38–4.29 (m, 1H), 4.17–4.06 (m, 1H), 3.98 (s, 3H), 3.96 (s, 3H), 3.59 (s, 3H), 3.29 (dd, *J* = 13.2, 4.3 Hz, 1H), 2.44 (s, 3H), 2.38–2.23 (m, 2H), 1.82 (dt, *J* = 11.0, 4.7 Hz, 1H) ppm. ^13^C-NMR (101 MHz, CDCl_3_) δ 182.3, 159.6, 153.5, 151.3, 150.4, 149.4, 146.7, 136.5, 134.8, 133.3, 129.9, 128.4, 126.0, 124.2, 121.4, 113.6, 61.5, 61.2, 61.2, 61.0, 60.8, 54.0, 35.1, 28.9, 15.2 ppm. ^19^F-NMR (379 MHz, CDCl_3_) δ −74.8 ppm. FT-IR (KBr pellet): 3221, 2938, 1735, 1610, 1543, 1464, 1411, 1349, 1325, 1283, 1244, 1161, 1100, 1081, 1021 cm^−1^. ESI-MS (*m*/*z*): [M + H]^+^ calcd. for 578, found 578, [M + 2 + H]^+^ calcd. 580, found 580, [M + Na]^+^ calcd. 600, found 600, [M + 2 + Na]^+^ calcd. 602, found 602.

##### Compound **9**

**Figure 9:**
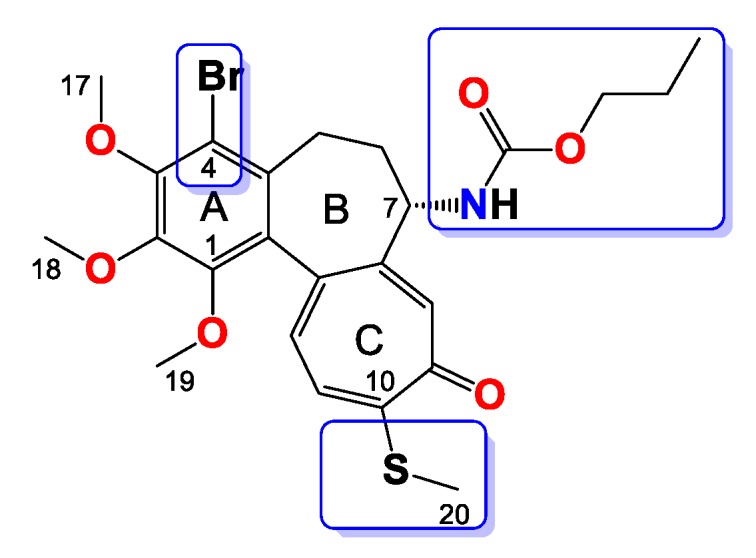
Structure of compound **9**.

Amorphous yellowish brown solid, yield 57 mg, 48%, MW = 538.5 g/mol (Figure 9). ^1^H-NMR (403 MHz, CDCl_3_) δ 7.33 (s, 1H), 7.21 (d, *J* = 10.3 Hz, 1H), 7.04 (d, *J* = 10.5 Hz, 1H), 5.39 (d, *J* = 7.3 Hz, 1H), 4.33 (dt, *J* = 12.6, 6.4 Hz, 1H), 3.98 (s, 3H), 3.96 (s, 3H), 3.95–3.90 (m, 2H), 3.61 (s, 3H), 3.27 (d, *J* = 9.1 Hz, 1H), 2.44 (s, 3H), 2.27 (dd, *J* = 7.1, 3.6 Hz, 2H), 1.69 (dd, *J* = 15.9, 5.1 Hz, 1H), 1.63–1.52 (m, 2H), 0.89 (t, *J* = 7.4 Hz, 3H) ppm. ^13^C-NMR (101 MHz, CDCl_3_) δ 182.3, 159.2, 155.6, 151.1, 150.4, 149.9, 146.6, 136.6, 134.5, 133.4, 130.1, 128.5, 125.8, 113.5, 66.9, 61.5, 61.4, 61.0, 53.4, 35.3, 29.0, 22.1, 15.2, 10.2 ppm. FT-IR (KBr pellet): 3300, 2937, 1717, 1608, 1549, 1463, 1410, 1348, 1324, 1288, 1245, 1153, 1083, 1020 cm^−1^. ESI-MS (*m*/*z*): [M + Na]^+^ calcd. 560, found 560, [M + 2 + Na]^+^ calcd. 562, found 562.

##### Compound **10**

**Figure 10:**
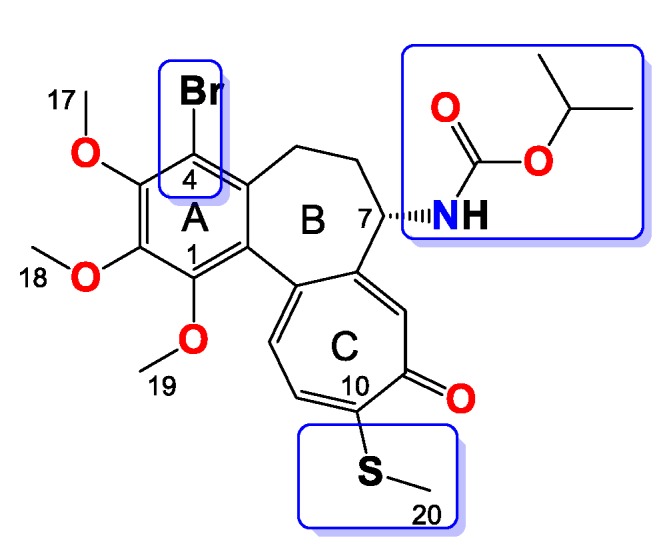
Structure of compound **10**.

Amorphous yellowish brown solid, yield 50 mg, 42%. MW = 538.5 g/mol (Figure 10). ^1^H-NMR (403 MHz, CDCl_3_) δ 7.29 (s, 1H), 7.19 (d, *J* = 10.3 Hz, 1H), 7.02 (d, *J* = 10.4 Hz, 1H), 5.15 (d, *J* = 6.7 Hz, 1H), 4.77 (dp, *J* = 12.5, 6.2 Hz, 1H), 4.36–4.26 (m, 1H), 3.97 (s, 3H), 3.95 (s, 3H), 3.60 (s, 3H), 3.29–3.23 (m, 1H), 2.43 (s, 3H), 2.25 (dd, *J* = 7.3, 3.9 Hz, 2H), 1.71–1.62 (m, 1H), 1.16 (dt, *J* = 16.2, 8.1 Hz, 6H) ppm. ^13^C-NMR (101 MHz, CDCl_3_) δ 182.4, 159.2, 155.0, 151.1, 150.4, 149.9, 146.6, 136.6, 134.5, 133.4, 130.1, 128.5, 125.8, 113.5, 68.7, 61.5, 61.4, 61.0, 53.3, 35.4, 29.0 22.1, 22.1, 15.2. FT-IR (KBr pellet): 3328, 2936, 1715, 1609, 1550, 1464, 1411, 1348, 1323, 1286, 1245, 1153, 1111, 1084, 1021 cm^−1^. ESI-MS (*m*/*z*): [M + Na]^+^ calcd. 560, found 560, [M + 2 + Na]^+^ calcd. 562, found 562.

##### Compound **11**

**Figure 11:**
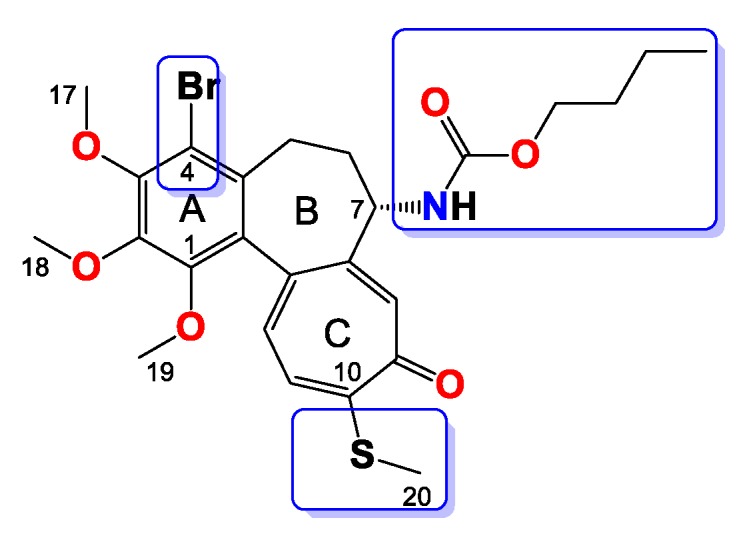
Structure of compound **11**.

Amorphous brownish solid, yield 54 mg, 44%. MW = 551.1 g/mol (Figure 11). ^1^H-NMR (403 MHz, CDCl_3_) δ 7.33 (s, 1H), 7.21 (d, *J* = 10.3 Hz, 1H), 7.04 (d, *J* = 10.4 Hz, 1H), 5.40 (d, *J* = 7.3 Hz, 1H), 4.38–4.27 (m, 1H), 3.98 (s, 3H), 3.96 (s, 3H), 3.94–3.90 (m, 2H), 3.61 (s, 3H), 3.27 (d, *J* = 8.8 Hz, 1H), 2.44 (s, 3H), 2.27 (dd, *J* = 7.1, 3.5 Hz, 2H), 1.71 (dd, *J* = 19.8, 11.1 Hz, 1H), 1.56 (ddd, *J* = 28.3, 14.4, 7.3 Hz, 2H), 1.32 (dt, *J* = 14.7, 7.4 Hz, 2H), 0.93–0.86 (m, 3H) ppm. ^13^C-NMR (101 MHz, CDCl_3_) δ 182.3, 159.2, 155.6, 151.1, 150.4, 149.9, 146.6, 136.6, 134.5, 133.4, 130.1, 128.5, 125.8, 113.5, 65.1, 61.5, 61.4, 61.0, 53.4, 35.3, 30.8, 28.9, 19.0, 15.2, 13.7 ppm. FT-IR (KBr pellet): 3288, 2935, 1717, 1608, 1548, 1463, 1410, 1347, 1325, 1246, 1154, 1083, 1020 cm^−1^. ESI-MS (*m*/*z*): [M + H]^+^ calcd. for 552, found 552, [M + 2 + H]^+^ calcd. 554, found 554, [M + Na]^+^ calcd. 574, found 574, [M + 2 + Na]^+^ calcd. 576, found 576.

##### Compound **12**

**Figure 12:**
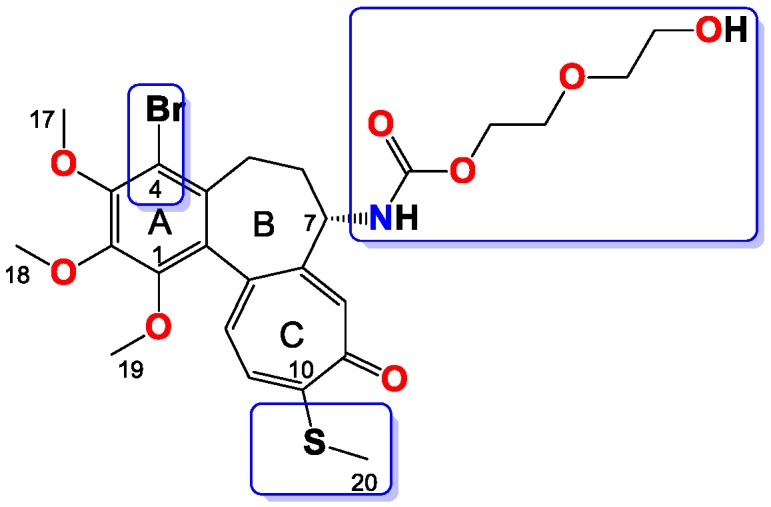
Structure of compound **12**.

Amorphous orangish solid, yield 54 mg, 42%. MW = 584.5 g/mol (Figure 12). ^1^H-NMR (403 MHz, CDCl_3_) δ 7.36 (s, 1H), 7.22 (d, *J* = 10.3 Hz, 1H), 7.06 (d, *J* = 10.6 Hz, 1H), 5.97 (d, *J* = 7.2 Hz, 1H), 4.35–4.20 (m, 2H), 4.13–4.06 (m, 1H), 3.98 (s, 3H), 3.96 (s, 3H), 3.78–3.70 (m, 2H), 3.65 (dd, *J* = 10.0, 5.3 Hz, 2H), 3.62–3.57 (m, 5H), 3.27 (d, *J* = 8.7 Hz, 1H), 2.44 (s, 3H), 2.27 (dd, *J* = 6.6, 3.5 Hz, 2H), 1.80–1.70 (m, 1H) ppm. ^13^C-NMR (101 MHz, CDCl_3_) δ 182.4, 159.3, 155.4, 151.2, 150.4, 150.2, 146.6, 136.8, 134.7, 133.4, 130.0, 128.3, 126.1, 113.5, 72.4, 69.1, 64.2, 61.5, 61.4, 61.0, 53.7, 34.9, 28.9, 15.2 ppm. FT-IR (KBr pellet): 3285, 2936, 1718, 1607, 1546, 1463, 1410, 1348, 1324, 1249, 1137, 1081, 1020 cm^−1^. ESI-MS (*m*/*z*): [M + Na]^+^ calcd. 606, found 606, [M + 2 + Na]^+^ calcd. 608, found 608.

### 2.4. Antiproliferative Activity of Colchicine and Its Derivatives

Four human cancer cell lines and one murine normal cell line were used to evaluate antiproliferative activity of colchicine and its derivatives (**2**–**12**): human lung adenocarcinoma (A549), human breast adenocarcinoma (MCF-7), human colon adenocarcinoma cell lines sensitive and resistant to doxorubicin (LoVo) and (LoVo/DX) respectively, and also normal murine embryonic fibroblast cell line (BALB/3T3). The BALB/3T3 cell line was purchased from the American Type Culture Collection (ATCC, Manassas, VA, USA), A549 and MCF-7 cell lines—from European Collection of Authenticated Cell Cultures (Salisbury, UK). The LoVo cell line was purchased from the American Type Culture Collection (ATCC, Manassas, VA, USA), and LoVo/DX by courtesy of Prof. E. Borowski (Technical University of Gdańsk, Gdańsk, Poland). All the cell lines are maintained in the Institute of Immunology and Experimental Therapy (IIET), Wroclaw, Poland. The human lung adenocarcinoma cell line was cultured in a mixture of OptiMEM and RPMI 1640 (1:1) medium (IIET, Wroclaw, Poland), supplemented with 5% foetal bovine serum (GE Healthcare, Logan UT, USA) and 2 mM l-glutamine (Sigma-Aldrich, Merck KGaA, Saint Louis, MO, USA). Human breast adenocarcinoma cell line was cultured in mixture of Eagle’s medium (IIET, Wroclaw, Poland), supplemented with 10% fetal bovine serum, 2 mM l-glutamine, 8 μg/mL insulin, and 1% amino-acids (Sigma-Aldrich, Merck KGaA, Saint Louis, MO, USA). Human colon adenocarcinoma cell lines were cultured in mixture of OptiMEM and RPMI 1640 (1:1) medium (IIET, Wroclaw, Poland), supplemented with 5% fetal bovine serum (GE Healthcare, Logan, UT, USA), 2 mM l-glutamine, 1 mM sodium pyruvate (Sigma-Aldrich, Merck KGaA, Saint Louis, MO, USA) and 10 μg/100 mL doxorubicin for LoVo/DX (Sigma-Aldrich, Merck KGaA, Saint Louis, MO, USA). Murine embryonic fibroblast cells were cultured in Dulbecco medium (Life Technologies Limited, Paisley, UK), supplemented with 10% foetal bovine serum (GE Healthcare, Logan, UT, USA) and 2 mM glutamine (Sigma-Aldrich, Merck KGaA, Saint Louis, MO, USA). All culture media contained antibiotics: 100 U/mL penicillin and 100 μg/mL streptomycin (Polfa–Tarchomin, Warsaw, Poland). All cell lines were cultured during the entire experiment in a humid atmosphere at 37 °C and 5% CO_2_. Cells were tested for mycoplasma contamination by mycoplasma detection kit for conventional PCR: Venor GeM Classic (Minerva Biolabs GmbH, Berlin, Germany) and negative results was obtained. The procedure was repeated every year or in the case of less frequently used lines: after thawing.

#### 2.4.1. The Antiproliferative Assays In Vitro

Twenty-four h before adding the tested compounds, all cell lines were seeded in 96-well plates (Sarstedt, Nümbrecht, Germany) in appropriate media with 10^4^ cells per well. All cell lines were exposed to each tested agent at four different concentrations in the range 100–0.01 μg/mL for 72 h. Cells were also exposed to the reference drug cisplatin (Teva Pharmaceuticals Polska, Warsaw, Poland) and doxorubicin (Accord Healthcare Limited, Middlesex, UK). Additionally, all cell lines were exposed to DMSO (solvent used for tested compounds) (POCh, Gliwice, Poland) at concentrations corresponding to these present in tested agents’ dilutions. After 72 h sulforhodamine B assay (SRB) was performed [26].

#### 2.4.2. SRB

After 72 h of incubation with the tested compounds, cells were fixed in situ by gently adding 50 μL per well of cold 50% trichloroacetic acid TCA (POCh, Gliwice, Poland) and were incubated at 4 °C for one hour. Following, wells were washed four times with water and air dried. Next, 50 μL of 0.1% solution of sulforhodamine B (Sigma-Aldrich, Merck KGaA, Saint Louis, MO, USA) in 1% acetic acid (POCh, Gliwice, Poland) were added to each well and plates were incubated at room temperature for 0.5 h. After incubation time, unbound dye was removed by washing plates four times with 1% acetic acid whereas stain bound to cells was solubilized with 10 mM Tris base (Sigma-Aldrich, Merck KGaA, Saint Louis, MO, USA). Absorbance of each solution was read at Synergy H4 Hybrid Multi-Mode Microplate Reader (BioTek Instruments, Inc., Winooski, VT, USA) at the 540 nm wavelength.

Results are presented as mean IC_50_ (concentration of the tested compound, that inhibits cell proliferation by 50%) ± standard deviation. IC_50_ values were calculated in Cheburator 0.4, Dmitry Nevozhay software (version 1.2.0 software by Dmitry Nevozhay, 2004–2014, http://www.cheburator.nevozhay.com, freely available) for each experiment [27]. Compounds at each concentration were tested in triplicates in single experiment and each experiment was repeated at least three times independently. Results are summarized in Table 1. The Resistance Index (RI) was defined as the ratio of IC_50_ for a given compound calculated for resistant cell line to that measured for its parental drug sensitive cell line (Table 1).

### 2.5. Molecular Docking Simulations

A combination of different computational methods was used to explore ligand-tubulin interactions. The ligands structures first minimized then fully optimized based on the RHF/cc-pVDZ level of theory in GAMESS-US version 2010-10-01. Since there is no crystal structure available for human βІ tubulin (TBB5_HUMAN), we obtained its sequence from UniProt (ID: Q13509). We used the tubulin structure 1SA0.pdb as a template to construct the homology model for βІ tubulin using MOE2015. We then docked the colchicine library to the protein using the Autodock4 program [28] under flexible ligand and rigid receptor conditions (Table 2). To verify the computed binding free energies, we employed two accurate but computationally expensive methods, namely MM/PBSA and MM/GBSA.

## 3. Results

### 3.1. Chemistry

**Scheme 1:**
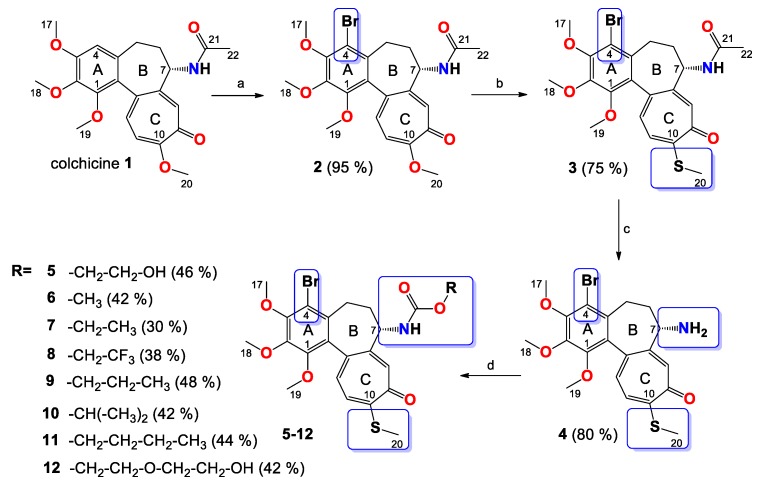
Synthesis of colchicine derivatives (**2**–**12**). Reagents and conditions: (**a**) N-bromosuccinimide (NBS), acetonitrile, room temperature; (**b**) MeOH/H_2_O, CH_3_SNa, room temperature; (**c**) 2M HCl, 90 °C, 72 h; (**d**) triphosgene, Et_3_N, respective alcohol, tetrahydrofuran (THF), 0 °C → room temperature.

In the present paper, we report the synthesis and spectroscopic analysis of a series of eight novel triple-modified colchicine derivatives (**5**–**12**). As shown in Scheme 1, 4-bromocolchicine (**2**), 4-bromothiocolchicine (**3**), and 4-bromo-*N*-deacetylthiocolchicine (**4**) were prepared according to previously described methods [22,24,25]. Compound **4** became the starting material for the synthesis of triple-modified colchicine derivatives. Compounds **5**–**12** were readily available from **4** by treatment with triphosgene as an activating reagent in the presence of triethylamine and the excess of respective alcohol or glycol in dry THF with yields from 30% to 48%. The structures of all products **2**–**12** were determined using the ESI-MS, FT-IR, ^1^H and ^13^C-NMR methods. Spectra are shown in Supplementary Materials (Figures S1–S23) and the results are also presented in the Materials and Methods section. All the spectroscopic and mass spectrometry data presented in Supplementary Materials confirm the structures of the studied compounds.

In the ^13^C-NMR spectra of the **2**, a resonance for the C-4 carbon atom of the A aromatic ring was observed at 113.5 ppm, while in **1** it was observed at 107.3 ppm. After the introduction of thiometyl group in C-10 positions shifts of the signal for the C-20 carbon atom in compound **3** were observed at 15.2 ppm, while in unmodified **1** as well as **2** shifts of the signal for the C-20 carbon atom were observed in the range 56.1–56.5 ppm. In the ^13^C-NMR spectra of the **4** signals for the acetyl group, which were observed at 170.0 and 22.9 ppm in compound **3**, had disappeared. In carbamates (**5**–**12**), shifts of the signal for the carbamate carbon atom were observed in the range 153.5–156.0 ppm.

### 3.2. In Vitro Determination of Drug-Induced Inhibition of Human Cancer Cell Line Growth

The eight triple-modified colchicine derivatives (**5**–**12**), three other colchicine derivatives (**2**–**4**), and starting material (**1**) were evaluated for their in vitro antiproliferative effect on normal and cancerous cells. Each compound was tested on four human cancer cell lines, including one cell line displaying various levels of drug resistance, namely human lung adenocarcinoma (A549), human breast adenocarcinoma (MCF-7), human colon adenocarcinoma cell line (LoVo) and doxorubicin-resistant subline (LoVo/DX). The antiproliferative effect was also studied on the normal murine embryonic fibroblast cell line (BALB/3T3) for better evaluation of cytotoxic activity of the compounds studied. The mean values IC_50_ ± SD of the tested compounds are summarized in Table 1. To evaluate the agents’ activity against the cells with MDR (multidrug resistance) phenotype, one drug resistant cancer cell line, i.e., LoVo/DX, was tested and the resistance index values (RI) were calculated (see Table 1). The RI values indicate how many times more resistant the subline was in comparison to its parental cell line.

All of the colchicine derivatives (**2**–**12**) showed higher antiproliferative activity against the A549 cell line than the commonly used cytostatic agents—doxorubicin (IC_50_ = 0.258 μM) and cisplatin (IC_50_ = 6.367 μM), as well as **1** itself (IC_50_ = 0.125 μM). The triple-modified colchicine derivatives (**5**–**12**) exhibited very significant activity with IC_50_ values ranging from 0.010 to 0.095 μM. As many as five of the compounds tested, including three of the triple-modified derivatives (**6**, IC_50_ = 0.013 μM; **7**, IC_50_ = 0.018 μM; **9**, IC_50_ = 0.027 μM), exhibited better activity against the MCF-7 cell line than **1** (IC_50_ = 0.054 μM). All of the colchicine derivatives (**2**–**12**) as well as **1** showed higher antiproliferative activity against the MCF-7 cell line than doxorubicin (IC_50_ = 0.386 μM) and cisplatin (IC_50_ = 10.70 μM). As many as nine of the colchicine derivatives (except **4** and **12**) showed better activity against the LoVo cell line, with IC_50_ values in the range of 0.007–0.085 μM, than **1** (IC_50_ = 0.108 μM) and doxorubicin (IC_50_ = 0.092 μM). All of the colchicine derivatives (**2**–**12**) as well as **1** showed higher antiproliferative activity against the LoVo cell line than cisplatin (IC_50_ = 4.37 μM).

The data presented in Table 1 show that all of the studied compounds including unmodified colchicine and the two reference anticancer drugs less effectively inhibited the proliferation of the resistant LoVo/DX cell line than the sensitive LoVo cell line. However, all of the colchicine derivatives (**2**–**12**) with IC_50_ values in the range of 0.050–1.550 μM as well as **1** (IC_50_ = 1.69 μM) showed higher antiproliferative activity against the LoVo/DX cell line than doxorubicin (IC_50_ = 4.75 μM) and cisplatin (IC_50_ = 5.70 μM). The calculated values of RI clearly confirmed that only compounds **10** and **11** (RI = 1.7 and 1.5, respectively) are able to efficiently overcome the drug resistance of the LoVo/DX cell line simultaneously showing very high antiproliferative activity (IC_50_ = 0.089 μM and 0.091 μM, respectively).

The values of the selectivity index (SI) were calculated to evaluate the toxicity of compounds against normal cells. The SI values calculated for A549, MCF-7, and LoVo cell lines were especially high (SI ≥ 4.3) for compounds **6**–**9** (except SI of **8** for the MCF-7 cell line). These values were much higher than the SI values of commonly used drugs, such as doxorubicin and cisplatin. High SI values result from large differences between the cytotoxicity against cancer and normal cells and this means that cancer cells will be killed at a higher rate than normal ones. Moreover, compounds **6**–**9** showed very high antiproliferative activity against A549, MCF-7, and LoVo cell lines, which is expressed by very low IC_50_ values (IC_50_ = 0.010–0.030; 0.013–0.027 (without **8**), and 0.007–0.018 for A549, MCF-7, and LoVo, respectively).

### 3.3. Molecular Docking: In Silico Determination of Drug-Induced Inhibition of βІ Tubulin

βІ tubulin, one of the subunits of microtubules in the cytoskeleton of eukaryotic cells, is a well-known and well-studied target for chemotherapeutic drugs selected to inhibit the growth and proliferation of cancer cells. The computational evaluation of binding energies between drug candidates and βІ tubulin performed by docking is a fast and inexpensive prediction method to investigate and rank the ability to arrest cancer cell proliferation by new compounds, which inhibit microtubule assembly. Here, the binding energies of new colchicine derivatives were calculated by docking the referred compounds to the colchicine-binding pocket of βІ tubulin.

A combination of different computational methods was used to explore ligand-tubulin interactions. The ligand structures were first energy-minimized, then fully optimized based on the RHF/cc-pVDZ level of theory implemented in the software package GAMESS-US, version 2010-10-01. Since there is no crystal structure for human βІ tubulin (UniProt ID: P07437) available in the Protein Data Bank (PDB), the bovine tubulin structure 1SA0.pdb was used as a template to construct the homology model for human βІ tubulin using the software package MOE2015. Autodock4 program under flexible ligand and rigid receptor conditions was also used to dock the small library of investigated colchicine derivatives to the target protein structure (see Table 2). AutoDock4 software is designed to predict how ligands bind to a receptor of a known 3D structure and it consists of two main modules: (1) autodock, which performs the docking of the ligand to a set of grids describing the target protein; and (2) auto grid, which pre-calculates these grids.

Based on our in silico calculations, **11**, **7**, **6**, and **3** show the strongest binding energies of −9.25, −9.20, −8.99, and −8.60 kcal/mol, respectively. Note that two Met 259 and Lys 352 residues in the colchicine binding site of βІ tubulin interact with C-20 (sidechain donor) and oxygen of carbonyl (sidechain acceptor) on ring C of the new colchicine derivatives, respectively. The diagrams depicting these interactions can be found in Table 2.

The evaluation of IC_50_ values in a cell-based assay involves interactions of the tested compounds with all tubulin expressed by the cell and βІ tubulin isotype is not the only isotype of tubulin expressed, although it is expected to be one of the most abundant ones. Even though both normal and cancer cells in humans contain the same tubulin isotypes, their expression levels and distribution of tubulin isotypes in each of the cell lines are different. In particular, the most abundant isotype in most tumors is βІ isotype (*TUBB*) followed by, βІVb (*TUBB4B*), βІIa (*TUBB2A*), βV (*TUBB6*), and βІII (*TUBB3*), with 47%, 38%, 8.9%, 3.1%, and 2.2%, respectively, and then βІVa (*TUBB4*), βІIb (*TUBB2B*), and βVI (*TUBB1*) with levels below 0.5% of the total expressed β tubulins. Since The expression levels of tubulin isotypes in each individual cell lines are unique, the binding energies for each of these isotypes would differ and affect the overall response to cytotoxic agents and make the computational prediction fairly complex [29]. To quantify the assumption that isotype expression levels correlate with cytotoxicity of the compounds acting on these isotypes of tubulin, the same docking simulation method was applied between the novel colchicine derivatives and βІIa (UniProt ID: Q13885), βІIb (UniProt ID: Q9BVA1), βІII (UniProt ID: Q13509), βІVa (UniProt ID: P04350), βІVb (UniProt ID: P68371), and βVI (UniProt ID: Q9H4B7) tubulin isotypes.

### 3.4. Linear Regression with Two Independent Variables

Following the docking simulations, in order to determine the level of correlation between experiment and computational simulations, we have calculated the linear regression coefficients between experimental values of IC_50_ and computational prediction for different β tubulin isotypes. To have a more realistic correlation coefficient, a logarithmic value of solubility of the compounds, the Moriguchi octanol-water partition coefficients (MLogP), were calculated using a software package called ADMET Predictor 8.0 (ADMET Predictor, Simulations Plus, Lancaster, CA, USA) and taken into account for predicting the biopharmaceutic properties like permeability and the understanding of transport mechanisms of the drugs in vivo [30]. The partition coefficient is also a useful factor in estimating and comparing the distribution of the drugs within the cells, organs, and the body.

A very good correlation of 0.66 and 0.84 involving log IC_50_ for LoVo and LoVo/DX cell lines, respectively, was the result of linear regression analysis with two independent variables, namely the compounds’ MlogP values and the other being the binding free energies of our compounds and the tubulin βІ isotype. These two cell lines have also a good correlation of 0.72 and 0.80 with the binding free energies of the compounds to the βІVb isotype, respectively. MlogP and the experimental IC_50_ values of 4-bromocolchicine based series are listed in Table 3, Supplementary Materials Figures S24 and S25. For the A549 cell line, the regression coefficients found were 0.43, 0.43, and 0.51 with binding free energies for βІ, βІVb, and βІVa isotypes, respectively. The reported regression coefficient for the A549 cell line is still acceptable, while its value for the MCF-7 and BALB/3T3 cell lines is very low (see Table 3). The reason for the latter discrepancy between computation and experiment may be due to additional biological factors, such as the upregulation of MDR proteins that act as efflux pumps and prevent the drugs from exerting their cytotoxic action. Another possibility could involve off-target interactions.

To further investigate the accuracy of the reported linear regressions between our computational results obtained using the docking method and the experimentally generated IC_50_ values, more accurate but also more time-consuming computational methods were applied to calculate the binding free energies between the novel colchicine derivatives and β tubulin isotypes. This analysis is described below.

### 3.5. MM/PBSA and MM/GBSA: In Silico Determination of Drug-Induced Inhibition of β Tubulin Isotypes

The molecular operating environment (MOE) was used to build a 3D model for the α-βI tubulin heterodimer [31]. Human tubulin protein sequences were obtained from UniProt [32]. The sequence corresponding to the gene *TUBA1A* (UniProt ID: Q71U36) was chosen as a reference sequence for human α-tubulin whereas gene *TUBB* associated to βI isoform (UniProt ID: P07437) was chosen for human β-tubulin. Homology modeling was performed using MOE by setting the number of generated models to 10 and by selecting the final model based on MOE’s generalized born/volume integral (GB/VI) scoring function. As a template, the crystallographic structure of α-βIIB tubulin isotype complexed with colchicine (PDB ID: 1SA0) was used [33]. During the modeling, cofactors including GTP, GDP, colchicine, and the magnesium ion located at the interface between α- and β-monomers were kept as part of the environment and included in the refinement step. The final model was eventually protonated at neutral pH and minimized using a MOE’s built-in protocol.

**Figure 13:**
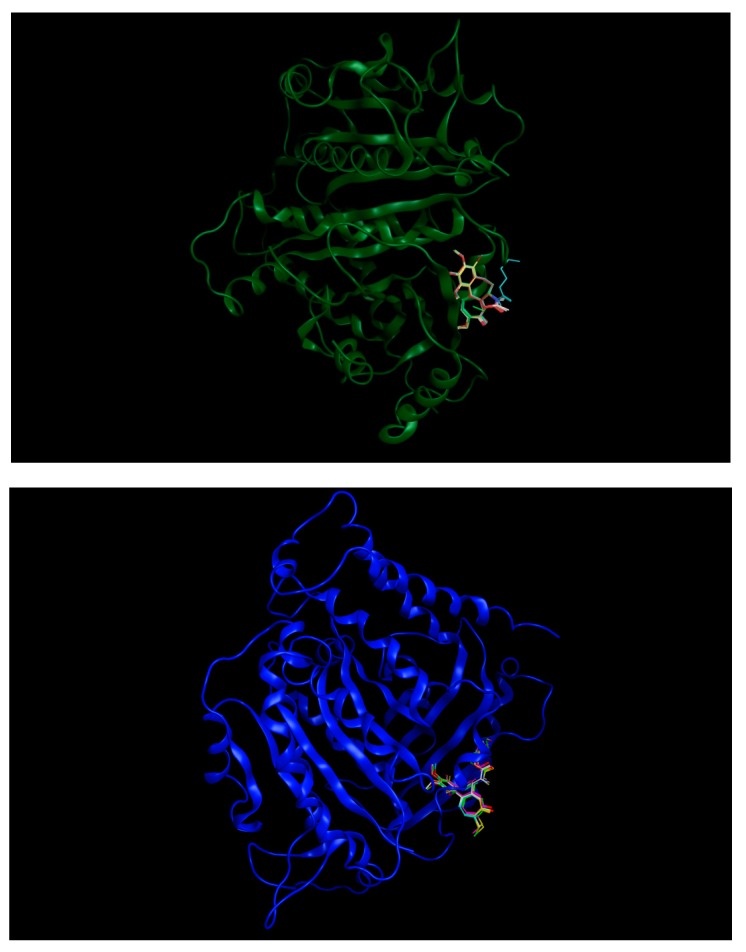
Tubulin βI structure in 3D with (**Top**) 5 colchicine derivatives with binding energies of −8.40 kcal/mol and higher, and (**Bottom**) with 6 colchicine derivatives with binding energies lower than −8.40 kcal/mol.

In order to equilibrate our model and get representative conformations, molecular dynamics (MDs) simulations were run using Amber14 [34]. Amber’s antechamber utility was applied to generate MD parameters—e.g., partial charges, force constants, etc.—for the four cofactors from the Gasteiger charge method. Amber’s tleap program was applied to solvate the system in TIP3P water. Minimization of the structure was carried out in two steps, both using the steepest descent and conjugate gradient methods successively. First, minimization was done during 2 ps on solvent atoms only, by restraining the protein-ligand complex. Next, minimization was run without the restraint during 10 ps. The structure was then equilibrated in an NVT ensemble during 20 ps and in an NPT ensemble during 40 ps setting the temperature to 298 K and the pressure to 1 bar. Finally, MD production was run for 70 ns. The root-mean-square deviation (RMSD) of both the entire tubulin structure and the colchicine binding site were found to plateau after 40 ns (see Figure 13).

Clustering analysis of the last 30 ns of the generated MD trajectory was carried out using Amber’s CPPTRAJ program [35] to identify representative conformations of the tubulin dimer. Clustering was done via a hierarchical agglomerative using the RMSD of atoms in the colchicine binding site as a metric. An RMSD cutoff of 1.0 Å was set to differentiate the clusters. From clustering analysis, three representative structures of the tubulin dimer were found. The structures were further used as a rigid target for the screening of 4-bromocolchicine derivatives.

Docking of 4-bromocolchicine derivatives was performed using the AutoDock Vina [36] program, which makes use of an iterated local search global optimizer as a searching method. The Vina scoring function combines aspects from knowledge-based and empirical potentials. Tested on the same set used for Autodock4, Vina enabled to identify the correct binding pose in 78% of the cases as compared to 53% for Autodock4 [37]. For our docking simulations, a cubic box with size 30.0 Å centered at the center of mass of the bound colchicine was used. All cofactors, namely, GTP, GDP, colchicine, and the magnesium ion were removed during docking while the target was kept rigid. For every compound, docking was run separately on each of the three tubulin representative structures obtained from clustering of the MD trajectory. Every generated pose was energy-minimized using Amber14 by keeping the protein fixed and was re-scored using the Vina software. For each compound/protein-structure pair, the pose with the best score was identified and used as an initial configuration for molecular mechanics Poisson–Boltzmann surface area MM/PBSA computations.

The MM/PBSA technique was used to calculate the free energy associated with the binding of 4-bromocolchicine derivatives [38,39]. This method combines molecular mechanics with continuum solvation models. The binding free energy is estimated as

(1) $$\Delta G_{bind}=\langle \Delta E_{MM}\rangle -T\Delta S+\Delta G_{solv},$$

where $\langle \Delta E_{MM}\rangle -T\Delta S$ can be regarded as the change in the free energy of the system in vacuum (gas phase); it includes the change in the molecular mechanics energy $\langle \Delta E_{MM}\rangle =\langle E_{MM}\rangle {}_{bound}-\langle E_{MM}\rangle {}_{unbound}$ and the change in the conformational entropy ΔS due to the binding. Since our goal was to compare the binding free energy of similar compounds derived from colchicine, ΔS was not estimated when calculating $\Delta G_{bind}$ as each compound is assumed to provide comparable ΔS values. $\Delta G_{solv}$ stands for the difference of solvation free energies due to the binding, which is given as $\Delta G_{solv}=\Delta G_{solv}^{complex}-\Delta G_{solv}^{lig}-\Delta G_{solv}^{prot}$ where every term on the right-hand side is given as the sum of polar and nonpolar contributions. The polar parts are obtained by solving the Poisson–Boltzmann (PB) equation or by using the generalized-born (GB) model—resulting in the MM/GBSA method, whereas the nonpolar terms are estimated from a linear relation to the solvent accessible surface area (SASA). The values of $\langle \Delta E_{MM}\rangle$ and $\Delta G_{solv}$ are generally computed as ensemble averages requiring a short MD trajectory of the solvated complexed system as input of the MM/PBSA method. In the present case, a 1 ns-duration MD trajectory was run in TIP3P water using Amber14, for every top pose generated at the end of the docking step. The MM/PBSA and MM/GBSA calculations were performed on a subset of 200 frames collected at regular time intervals from the trajectory. For PB calculations, an ionic strength of 0.0 nM (istrng = 0.0) and a solvent probe radius of 1.6 Å (prbrad = 1.6) were used. For GB calculations, the igb parameter was set to 5 that corresponds to a modified GB model equivalent to model II in reference [40]. For each of the tested compounds, the best PB and GB score out of the three trajectories associated with the three representative structures of the tubulin dimer were collected and reported in Table 4.

### 3.6. Linear Regression with Two Independent Variables

Following the MM/PBSA simulations, we have calculated the linear regression coefficients between experiment and computational simulations of different β tubulin isotypes. The result of linear regression with two independent variables between the binding free energies of βІVb isotypes, MlogP and IC_50_ for the LoVo and LoVo/DX cell lines is that very good correlation coefficients were found, namely 0.78 and 0.67, respectively. Also, reasonably good correlation of 0.65 and 0.54 was obtained with the binding free energies of βІ isotype for these two cell lines, respectively. These correlation coefficients are also consistent with the correlation coefficients calculated using the docking binding energies and these cell lines except for LoVo/DX and βІ which were lower but still acceptable. For the A549 cell line, the regression coefficients found were 0.62, 0.58, and 0.48 for the binding free energies with βІII, βІVb, and βІ isotypes, respectively. The reported regression coefficient for the A549 cell line was improved a little bit by using the more expensive method but the MM/PBSA method could not improve the correlation coefficient values for the MCF-7 and BALB/3T3 cell lines and they are still very low (Table 5).

The LoVo and LoVo/DX cell line correlation with βІ and βІVb isotypes 0.60, 0.66, 0.65, and 0.71 respectively. The result also shows a good correlation of 0.7 between binding energies for βV tubulin and LoVo/DX cell line. For, the regression coefficients of 0.55 and 0.51 with free energies of βI, the isotypes which showed for the A549 cell line were in the same range with docking and MM/GBSA results. There were no improvements for the correlation values for the MCF-7 and BALB/3T3 cell lines with the MM/GBSA simulations (Table 6).

The ranges of correlation coefficients between the IC_50_ values of all the cell lines and the corresponding binding free energies calculated with three different methods are the same. Reassuringly, the best correlation values from all three methods used were found for βI and βIVb tubulin isotypes, which are reported to be most abundant in these types of cells.

## 4. Discussion

We synthesized a series of novel triple-modified 4-bromothiocolchicine derivatives (**5**–**12**) as well as 4-bromocolchicine (**2**), 4-bromothiocolchine (**3**) and 4-bromo-*N*-deacetylthiocolcicine (**4**) and evaluated their biological activity according to the in vitro antiproliferative tests as well as in silico studies. Biological activity was evaluated on four human cancer cell lines and a normal murine embryonic fibroblast cell line. The results of our study have clearly showed that the cytotoxicity of almost all colchicine derivatives (**2**–**12**) is higher than the corresponding cytotoxicity of commonly used cytostatic agents—doxorubicin and cisplatin against A549, MCF-7, LoVo (except **4** and **12**) and LoVo/DX cancer cell lines. The majority of the derivatives also exhibit a higher cytotoxicity than unmodified colchicine. Particularly noteworthy are the compounds **6**–**9**, which show very high antiproliferative activity against A549, MCF-7 and LoVo cell lines, that is expressed by very low IC_50_ values at nanomolar concetrations (IC_50_ = 0.010–0.030; 0.013–0.027 (without **8**) and 0.007–0.018 μM for A549, MCF-7 and LoVo, respectively). Moreover, compounds **6**–**9** were demonstrated to be less toxic to normal murine fibroblast cells than the currently used anticancer drugs, such as cisplatin and doxorubicin, which is confirmed by particularly high selectivity index (SI) values calculated for A549, MCF-7, and LoVo cell lines are (SI ≥ 4.3, except SI of eight for MCF-7 cell line). The toxicity is a major challenge in designing a potential colchicine-based drug candidate. High SI values result from large differences between the cytotoxicity against cancer and normal cells and this means that cancer cells exposed to the same concentration of the compound will be killed at a higher rate than normal ones, which might lead to a potential colchicine-based drug candidate.

Based on our in silico calculations, **11**, **7**, **6**, and **3** show the strongest binding energies of −9.25, −9.20, −8.99 and −8.60 kcal/mol, respectively. Two Met 259 and Lys 352 residues in the colchicine binding site of βІ tubulin interact with C-20 (sidechain donor) and oxygen of carbonyl (sidechain acceptor) on ring C of the new colchicine derivatives, respectively. In order to determine the level of correlation between experiment and computational simulations, we have calculated the linear regression coefficients between IC_50_ values and the binding free energies involving the compounds and tubulin isotypes as well as the MlogP coefficients for these compounds. A very good correlation of 0.66 and 0.84 with log IC_50_ for LoVo and LoVo/DX cell lines, respectively, has been found. For the A549 cell line, the regression coefficient found is 0.43, still acceptable, while its value for the MCF-7 and BALB/3T3 cell lines is very low. This may be explained by a number of additional effects taking place in living cells compared to the computational simulations that focus only on the binding mode of the compounds to the target. Specifically, off-target interactions involving efflux pumps with different affinities for the individual compounds may explain the observed partial correlation between IC_50_ values and binding free energies. Additionally, differences in the solubility values and membrane permeability may have to be accounted for when ranking the various compounds in biological assays and comparing them to computational predictions based on binding affinity alone. We have additionally supported these findings with calculations using two very accurate methods of calculating the binding energies of ligands to proteins, namely MM/PBSA and MM/GBSA. The results using all three in silico approaches have been found consistent.

In short, these results confirm that a suitable chemical modification of colchicine aimed for an improved binding affinity to human tubulin ßI or ßIVb isotypes is a promising approach to finding highly biologically active and less toxic compounds. Some of the obtained compounds are suitable candidates for further tests (ex vivo, in vivo). The synthesis of new colchicine derivatives with diverse substituents is also a next step in developing structure-activity relationship (SAR) of colchicine-binding site inhibitors.

## Figures and Tables

**Table 1:** Antiproliferative activity of colchicine (**1**) and its derivatives (**2**–**12**) compared with antiproliferative activity of standard anticancer drugs doxorubicin and cisplatin and the calculated values of resistance index (RI) and selectivity index (SI) of tested compounds.

	Cancer Cells									Normal Cells
Compound	A549		MCF-7		LoVo		LoVo/DX			BALB/3T3
	IC_50_ (μM)	SI	IC_50_ (μM)	SI	IC_50_ (μM)	SI	IC_50_ (μM)	SI	RI	IC_50_ (μM)
**1**	0.125 ± 0.013	1.11	0.054 ± 0.028	2.57	0.108 ± 0.025	1.29	1.69 ± 0.28	0.08	15.65	0.139 ± 0.073
**2**	0.105 ± 0.008	1.35	0.027 ± 0.008	5.26	0.084 ± 0.021	1.69	1.550 ± 0.170	0.09	18.45	0.142 ± 0.073
**3**	0.010 ± 0.0001	10.3	0.015 ± 0.002	6.87	0.014 ± 0.004	7.36	0.135 ± 0.012	0.76	9.64	0.103 ± 0.089
**4**	0.115 ± 0.007	8.13	0.178 ± 0.020	7.08	0.125 ± 0.044	10.08	0.700 ± 0.088	1.80	5.60	1.260 ± 0.796
**5**	0.074 ± 0.009	1.41	0.057 ± 0.011	1.82	0.074 ± 0.019	1.41	1.010 ± 0.020	0.10	13.65	0.104 ± 0.043
**6**	0.010 ± 0.0001	6.60	0.013 ± 0.002	5.08	0.007 ± 0.002	9.43	0.050 ± 0.010	1.32	7.14	0.066 ± 0.031
**7**	0.012 ± 0.004	8.50	0.018 ± 0.002	5.67	0.011 ± 0.004	9.27	0.071 ± 0.010	1.44	6.45	0.102 ± 0.063
**8**	0.030 ± 0.021	4.60	0.055 ± 0.026	2.51	0.018 ± 0.010	7.67	0.074 ± 0.007	1.86	4.11	0.138 ± 0.010
**9**	0.012 ± 0.004	9.67	0.027 ± 0.007	4.30	0.011 ± 0.0001	10.55	0.072 ± 0.011	1.61	6.55	0.116 ± 0.009
**10**	0.089 ± 0.020	1.94	0.132 ± 0.017	1.31	0.054 ± 0.017	3.20	0.089 ± 0.026	1.94	1.65	0.173 ± 0.108
**11**	0.095 ± 0.005	1.54	0.125 ± 0.014	1.17	0.062 ± 0.013	2.35	0.091 ± 0.009	1.60	1.47	0.146 ± 0.014
**12**	0.093 ± 0.014	2.14	0.125 ± 0.015	1.08	0.281 ± 0.185	0.48	4.240 ± 1.330	0.03	15.09	0.135 ± 0.015
Doxorubicin	0.258 ± 0.044	0.64	0.386 ± 0.118	0.43	0.092 ± 0.018	1.80	4.75 ± 0.99	0.035	51.60	0.166 ± 0.074
Cisplatin	6.367 ± 1.413	0.61	10.70 ± 0.753	0.36	4.37 ± 0.73	0.89	5.70 ± 0.63	0.68	1.3	3.90 ± 1.50

The IC_50_ value is defined as the concentration of a compound at which 50% growth inhibition is observed. The SI (selectivity index) was calculated for each compound using the formula: SI = IC_50_ for normal cell line BALB/3T3/IC_50_ for respective cancerous cell line. A beneficial SI > 1.0 indicates a drug with efficacy against tumor cells greater than the toxicity against normal cells. The RI (resistance index) indicates how many times a resistant subline is chemo-resistant relative to its parental cell line. The RI was calculated for each compound using the formula: RI = IC_50_ for LoVoDX/IC_50_ for LoVo cell line. When RI is 0–2, the cells are sensitive to the compound tested, RI in the range 2–10 means that the cell shows moderate sensitivity to the drug tested, RI above 10 indicates strong drug-resistance.

**Table 2:** Calculated binding energies for the interactions between the new colchicine derivatives investigated in this paper and βІ tubulin, and also the values of the Moriguchi octanol-water partition coefficient, MlogP, calculated for the same colchicine derivatives.

Compound	Binding Energy (kcal/mol)	Interactions	MW	MlogP	Active Residues
**1**	−8.09	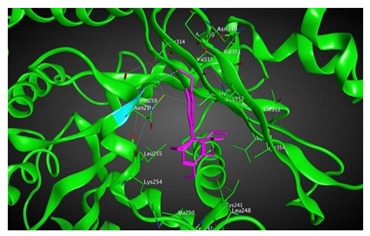	399.44	1.37	Asn258
**2**	−8.40	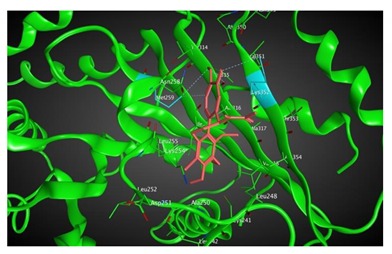	481.37	1.93	Met259 Asn258 Lys352
**3**	−8.60	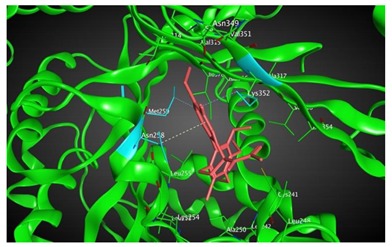	497.44	2.69	Met259 Lys352 Val315
**4**	−8.12	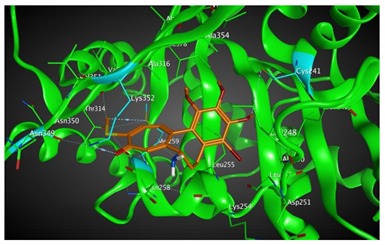	455.40	2.71	Asn349 Met259 Lys352 Cys241
**5**	−7.68	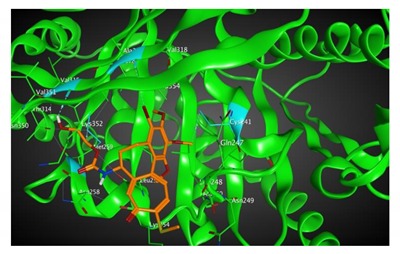	543.46	2.20	Cys241 Ala317 Val315 Met259
**6**	−8.99	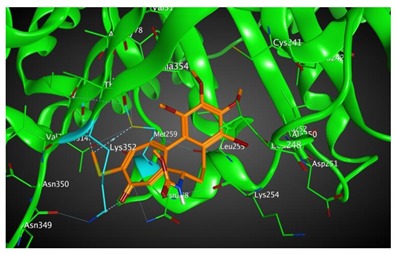	513.43	2.74	Met259 Lys352 Val315
**7**	−9.20	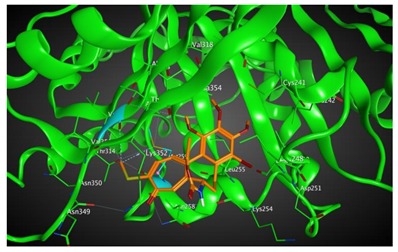	527.46	2.95	Met259 Val315
**8**	−7.61	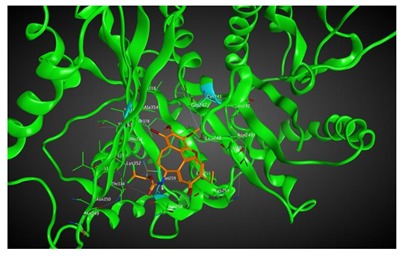	581.43	3.26	Cys241 Asn258 Ala317
**9**	−8.21	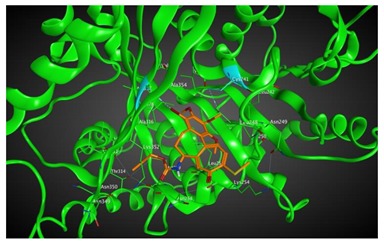	541.49	3.16	Cys241 Ala317
**10**	−8.06	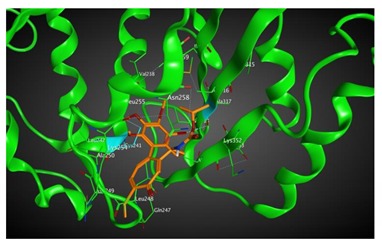	541.49	3.16	Cys241 Ala317
**11**	−9.25	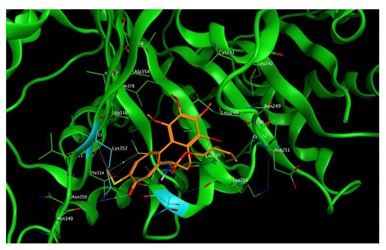	528.51	2.30	Lys352 Asn258 Val315
**12**	−7.92	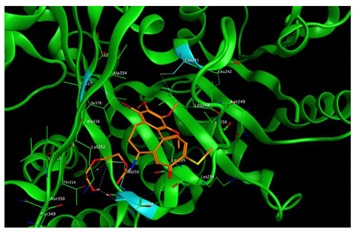	587.52	1.87	Ala317 Asn258 Met259 Cys241

**Table 3:** Linear regressions involving two independent variables (the binding free energy obtained from the docking method and MlogP) of the investigated colchicine derivatives versus log IC_50_ [μM] in different cancer cell lines.

		**MCF-7**	**LoVo**	**LoVo/DX**	**A549**	**BALB/3T3**
Linear regression of colchicine derivatives (R^2^)	βI	0.11	0.66	0.84	0.43	0.02
	βIIa	0.01	0.53	0.68	0.30	0.07
	βIIb	0.15	0.69	0.76	0.42	0.0004
	βIII	0.01	0.57	0.71	0.36	0.09
	βIVa	0.16	0.65	0.74	0.51	0.31
	βIVb	0.02	0.72	0.80	0.43	0.01
	βVI	0.03	0.56	0.66	0.38	0.09

**Table 4:** Binding energies were calculated by MM/PBSA and MM/GPSA methods.

Ligand	GBSA βI	PBSA βI	GBSA βIIa	PBSA βIIa	GBSA βIII	PBSA βIII	GBSA βIVb	PBSA βIVb	GBSA βVI	PBSA βVI
**1**	−40.45	−40.20	−40.83	−45.40	−33.53	−32.05	−39.53	−44.63	−43.07	−35.61
**2**	−36.57	−44.26	−46.99	−31.05	−33.09	−32.52	−44.79	−36.88	−46.81	−44.01
**3**	−33.97	−37.17	−33.04	−29.80	−27.66	−28.16	−34.17	−30.91	−33.5	−37.88
**4**	−37.12	−43.09	−36.51	−41.35	−34.78	−31.90	−40.18	−43.70	−39.14	−33.84
**5**	−36.80	−44.14	−33.28	−42.68	−29.91	−22.86	−34.98	−39.89	−37.87	−39.19
**6**	−37.20	−45.72	−38.78	−38.70	−29.35	−29.16	−30.74	−22.96	−34.44	−31.81
**7**	−40.29	−44.06	−39.28	−45.47	−28.69	−26.87	−40.99	−49.02	−41.90	−42.59
**8**	−48.15	−51.01	−40.07	−25.4	−45.89	−50.04	−47.43	−50.29	−50.68	−45.55
**9**	−54.12	−39.54	−58.30	−54.17	−42.75	−41.12	−41.59	−32.40	−61.45	−51.74
**10**	−58.57	−48.74	−60.92	−37.23	−61.92	−55.56	−58.22	−55.78	−59.39	−49.74
**11**	−46.77	−49.84	−41.34	−41.52	−29.28	−19.52	−43.05	−23.88	−45.26	−35.5
**12**	−52.71	−45.46	−55.36	−34.82	−38.95	−26.10	−53.97	−32.11	−50.28	−46.36

MM/PBSA—Molecular Mechanics Poisson-Boltzmann Surface Area, MM/GBSA—Molecular Mechanics Generalized Born Surface Area, PBSA—Poisson-Boltzmann Surface Area, GBSA—Generalized Born Surface Area.

**Table 5:** Linear regressions involving two independent variables (binding energy by MM/PBSA method and MlogP) of the investigated colchicine derivatives versus log IC_50_ (μM) in different cancer cell lines.

		**MCF-7**	**LoVo**	**LoVo/DX**	**A549**	**BALB/3T3**
Linear regression of colchicine derivatives (R^2^)	βI	0.18	0.65	0.54	0.48	0.0006
	βIIa	0.09	0.57	0.56	0.41	0.0006
	βIII	0.31	0.61	0.40	0.62	0.03
	βIVb	0.22	0.78	0.67	0.58	0.03
	βVI	0.17	0.64	0.67	0.47	0.0005

**Table 6:** Linear regressions involving two independent variables (binding energy by MM/GBSA method and MlogP) of the investigated colchicine derivatives versus log IC50 (μM) in different cancer cell lines.

		**MCF-7**	**LoVo**	**LoVo/DX**	**A549**	**BALB/3T3**
Linear regression of colchicine derivatives (R^2^)	βI	0.21	0.60	0.65	0.55	0.0005
	βIIa	0.02	0.54	0.68	0.36	0.0004
	βIII	0.08	0.57	0.59	0.46	0.013
	βIVb	0.12	0.66	0.71	0.51	0.095
	βVI	0.04	0.58	0.70	0.37	0.013

## Supplementary Materials

The following are available online at http://www.mdpi.com/2073-4409/7/11/216/s1, Figure S1: The ^13^C-NMR spectrum of 2 in CDCl_3_. Figure S2: The ^1^H-NMR spectrum of 2 in CDCl_3_. Figure S3: The ^13^C-NMR spectrum of 3 in CDCl_3_. Figure S4: The ^1^H-NMR spectrum of 3 in CDCl_3_. Figure S5: The ^13^C-NMR spectrum of 4 in CDCl_3_. Figure S6: The ^1^H-NMR spectrum of 4 in CDCl_3_. Figure S7: The ^13^C-NMR spectrum of 5 in CDCl_3_. Figure S8: The ^1^H-NMR spectrum of 5 in CDCl_3_. Figure S9: The ^13^C-NMR spectrum of 6 in CDCl_3_. Figure S10: The ^1^H-NMR spectrum of 6 in CDCl_3_. Figure S11: The ^13^C-NMR spectrum of 7 in CDCl_3_. Figure S12: The ^1^H-NMR spectrum of 7 in CDCl_3_. Figure S13: The ^13^C-NMR spectrum of 8 in CDCl_3_. Figure S14: The ^1^H-NMR spectrum of 8 in CDCl_3_. Figure S15: The ^19^F-NMR spectrum of 8 in CDCl_3_. Figure S16. The ^13^C-NMR spectrum of 9 in CDCl_3_. Figure S17: The ^1^H-NMR spectrum of 9 in CDCl_3_. Figure S18: The ^13^C-NMR spectrum of 10 in CDCl_3_. Figure S19: The ^1^H-NMR spectrum of 10 in CDCl_3_. Figure S20: The ^13^C-NMR spectrum of 11 in CDCl_3_. Figure S21: The ^1^H-NMR spectrum of 11 in CDCl_3_. Figure S22: The ^13^C-NMR spectrum of 12 in CDCl_3_.Figure S23: The ^1^H-NMR spectrum of 12 in CDCl_3_. Figure S24: 3D plots of the linear regression results for the binding energies (BE) vs. MlogP vs. log IC_50_ [μM] values for LoVo cell line. Figure S25: 3D plots of the linear regression results for the binding energies (BE) vs. MlogP vs log IC_50_ [μM] values for LoVo/DX cell line.
