# Supplementary material for: Synthesis and Biological Evaluation of Novel Triple-Modified Colchicine Derivatives as Potent Tubulin-Targeting Anticancer Agents
Source: Cells. 2018 Nov 19;7(11):216. doi: 10.3390/cells7110216 (PMC6262455; doi:10.3390/cells7110216)
Supplement: Supplementary file 1 [file cells-07-00216-s001.pdf]

cells

Supplementary Materials

Synthesis and biological evaluation of novel triple-modified Colchicine derivatives as potent tubulin-targeting anticancer agents

Urszula Majcher[1], Greta Klejborowska[1], Magdalena Kaik[1], Ewa Maj[2], Joanna Wietrzyk[2], Mahshad Moshari[3], Jordane Preto[4], Jack A. Tuszynski[4,5] and Adam Huczyński[1,*]

[1] Department of Bioorganic Chemistry, Faculty of Chemistry, Adam Mickiewicz University, Umultowska 89b, 61-614 Poznan, Poland.
[2] Hirszfeld Institute of Immunology and Experimental Therapy, Polish Academy of Sciences, Rudolfa Weigla 12, 53-114 Wrocław, Poland.
[3] Depertment of Chemistry, University of Alberta, Edmonton, Alberta T6G 1Z2, Canada.
[4] Depertment of Oncology, University of Alberta, Edmonton, Alberta T6G 1Z2, Canada.
[5] DIMEAS, Politecnico di Torino, Corso Duca degli Abruzzi 24, Turin, Italy.
* Correspondence: adhucz@amu.edu.pl; Tel.: +48-61-829-1673

Supplementary Materials

Figure S1. The ^{13}C NMR spectrum of **2** in $CDCl_3$.
Figure S2. The ^{1}H NMR spectrum of **2** in $CDCl_3$.
Figure S3. The ^{13}C NMR spectrum of **3** in $CDCl_3$.
Figure S4. The ^{1}H NMR spectrum of **3** in $CDCl_3$.
Figure S5. The ^{13}C NMR spectrum of **4** in $CDCl_3$.
Figure S6. The ^{1}H NMR spectrum of **4** in $CDCl_3$.
Figure S7. The ^{13}C NMR spectrum of **5** in $CDCl_3$.
Figure S8. The ^{1}H NMR spectrum of **5** in $CDCl_3$.
Figure S9. The ^{13}C NMR spectrum of **6** in $CDCl_3$.
Figure S10. The ^{1}H NMR spectrum of **6** in $CDCl_3$.
Figure S11. The ^{13}C NMR spectrum of **7** in $CDCl_3$.
Figure S12. The ^{1}H NMR spectrum of **7** in $CDCl_3$.
Figure S13. The ^{13}C NMR spectrum of **8** in $CDCl_3$.
Figure S14. The ^{1}H NMR spectrum of **8** in $CDCl_3$.
Figure S15. The ^{19}F NMR spectrum of **8** in $CDCl_3$
Figure S16. The ^{13}C NMR spectrum of **9** in $CDCl_3$.
Figure S17. The ^{1}H NMR spectrum of **9** in $CDCl_3$.
Figure S18. The ^{13}C NMR spectrum of **10** in $CDCl_3$.
Figure S19. The ^{1}H NMR spectrum of **10** in $CDCl_3$.
Figure S20. The ^{13}C NMR spectrum of **11** in $CDCl_3$.
Figure S21. The ^{1}H NMR spectrum of **11** in $CDCl_3$.
Figure S22. The ^{13}C NMR spectrum of **12** in $CDCl_3$.
Figure S23. The ^{1}H NMR spectrum of **12** in $CDCl_3$.
Figure S24. 3D plots of the linear regression results for the binding energies (BE) vs MlogP vs log IC_{50} [μM] values for LoVo cell line.

cells

Figure S25. 3D plots of the linear regression results for the binding energies (BE) vs MlogP vs log IC_{50} [μM] values for LoVo/DX cell line.

.

cells

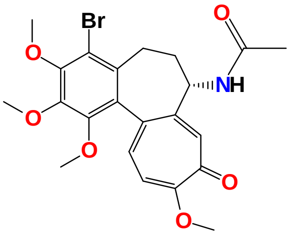

Chemical formula: $C_{22}H_{24}BrNO_6$, MW = 478.3 g/mol

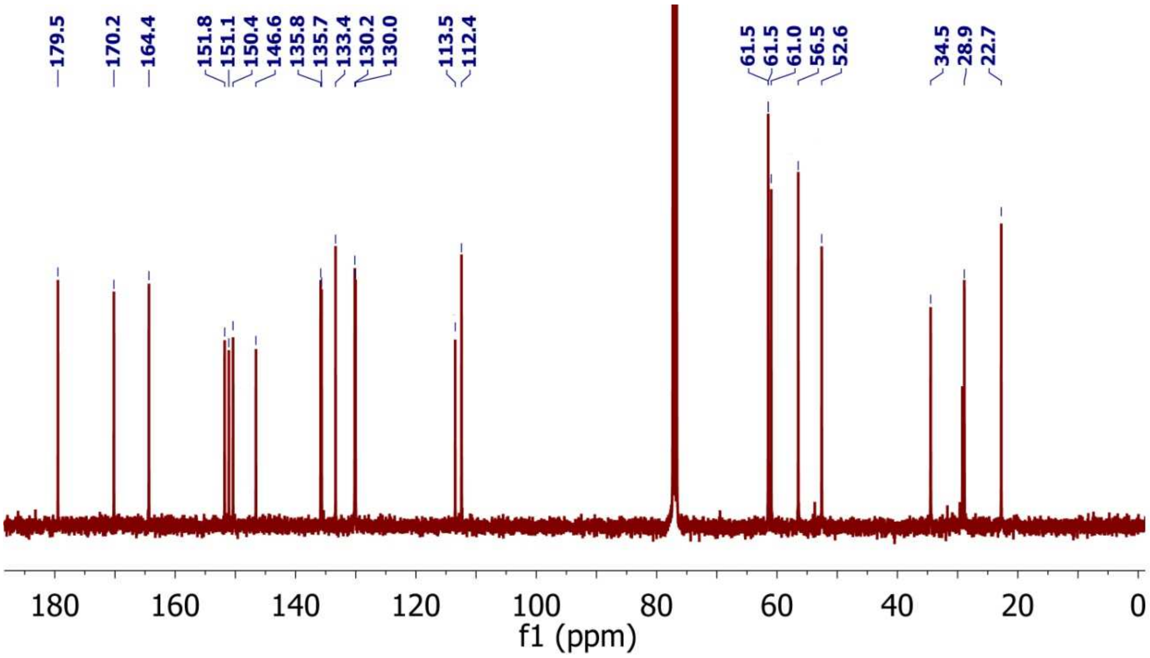

Figure S1. The ^{13}C NMR spectrum of **2** in $CDCl_3$.

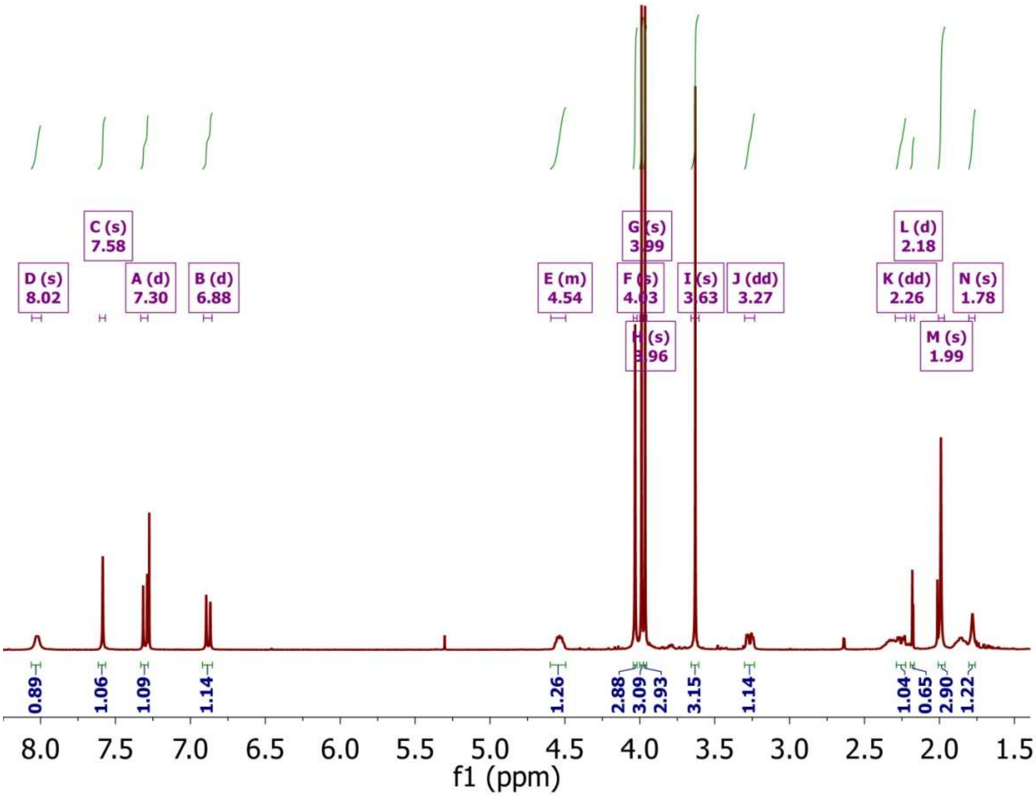

Figure S2. The ^{1}H NMR spectrum of **2** in $CDCl_3$.

cells

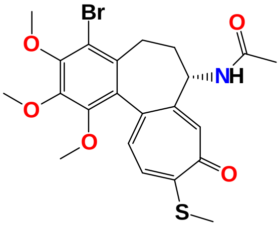

Chemical formula: $C_{22}H_{24}BrNO_5S$, MW = 494.4 g/mol

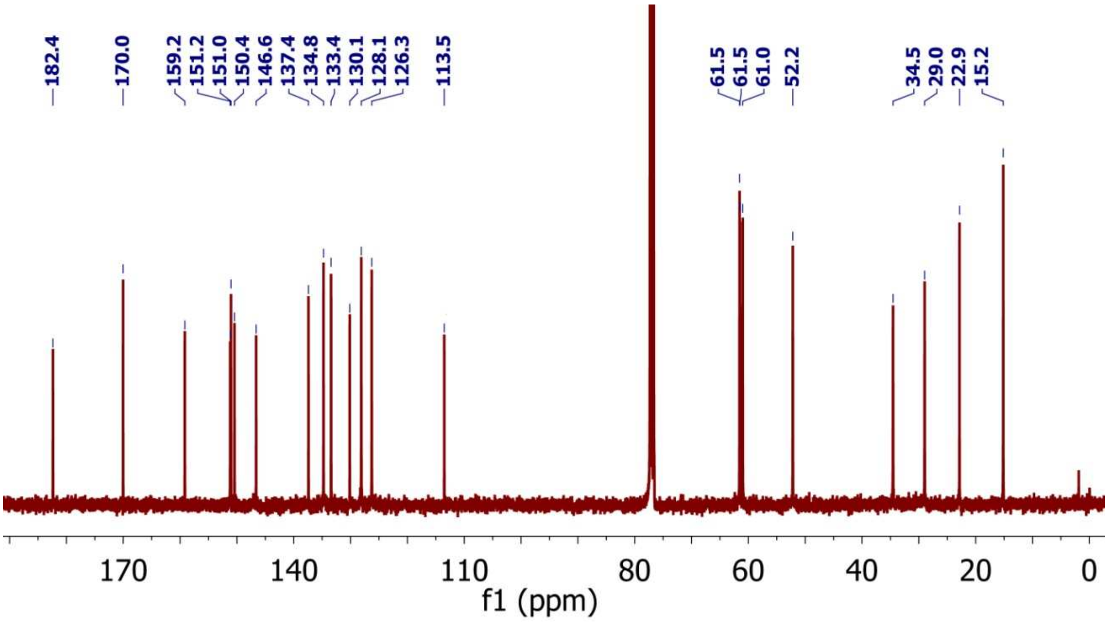

Figure S3. The ^{13}C NMR spectrum of **3** in $CDCl_3$.

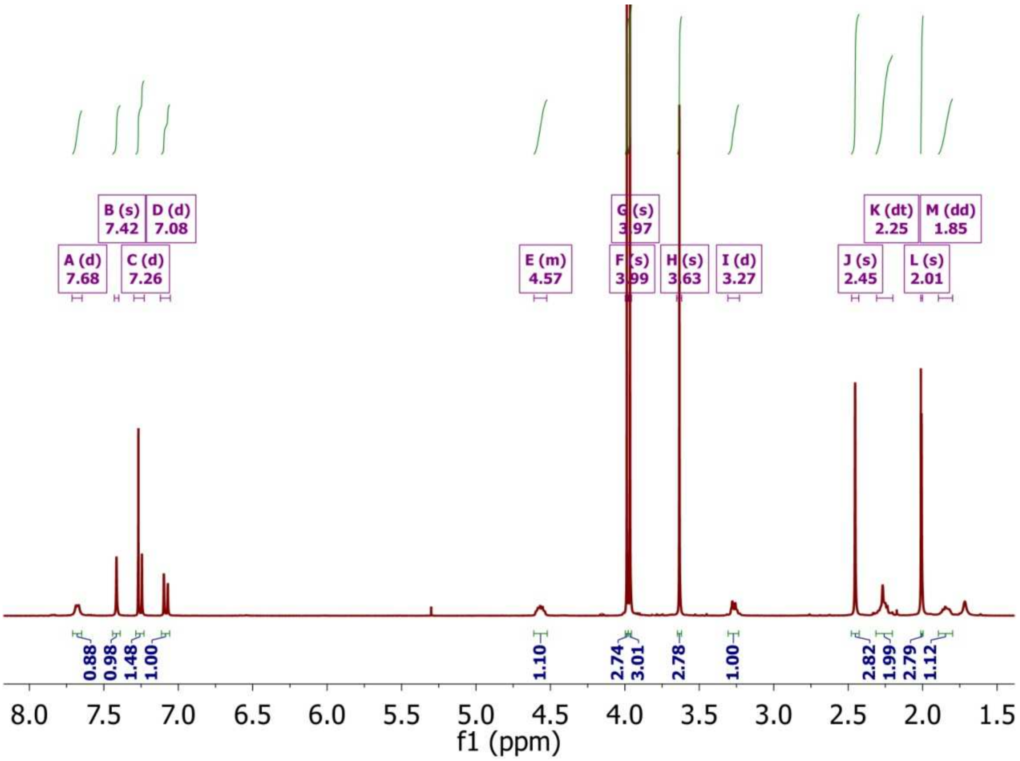

Figure S4. The ^{1}H NMR spectrum of **3** in $CDCl_3$.

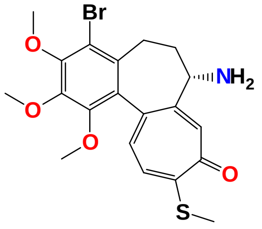

Chemical formula: $C_{20}H_{22}BrNO_4S$, MW = 452.4 g/mol

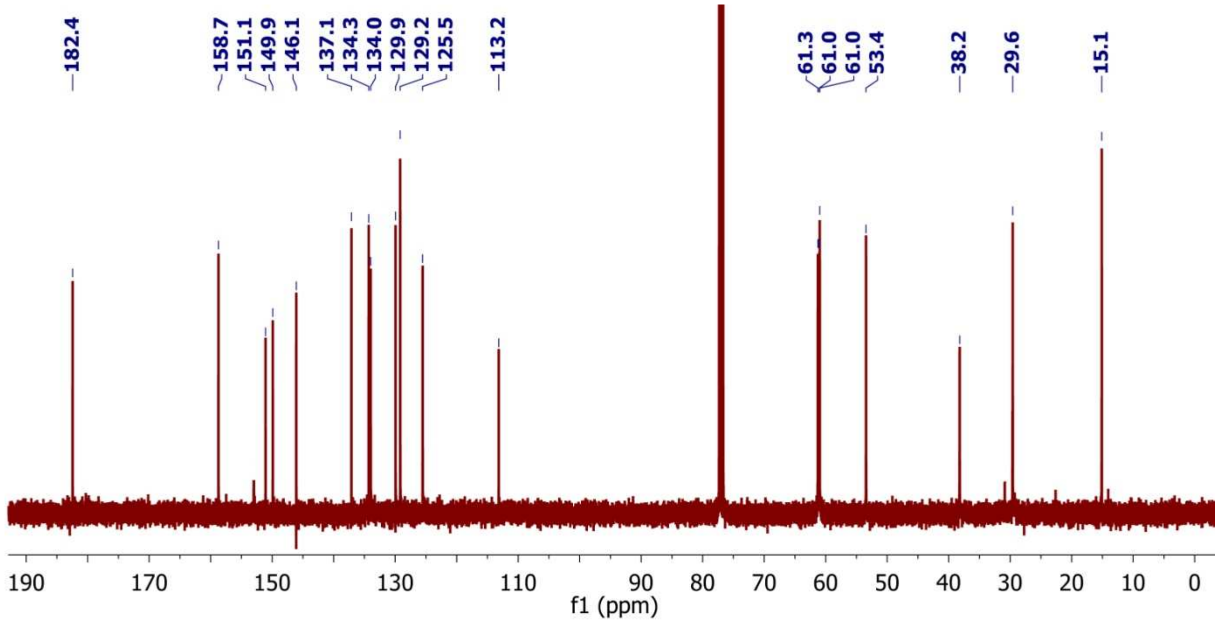

Figure S5. The ^{13}C NMR spectrum of **4** in $CDCl_3$.

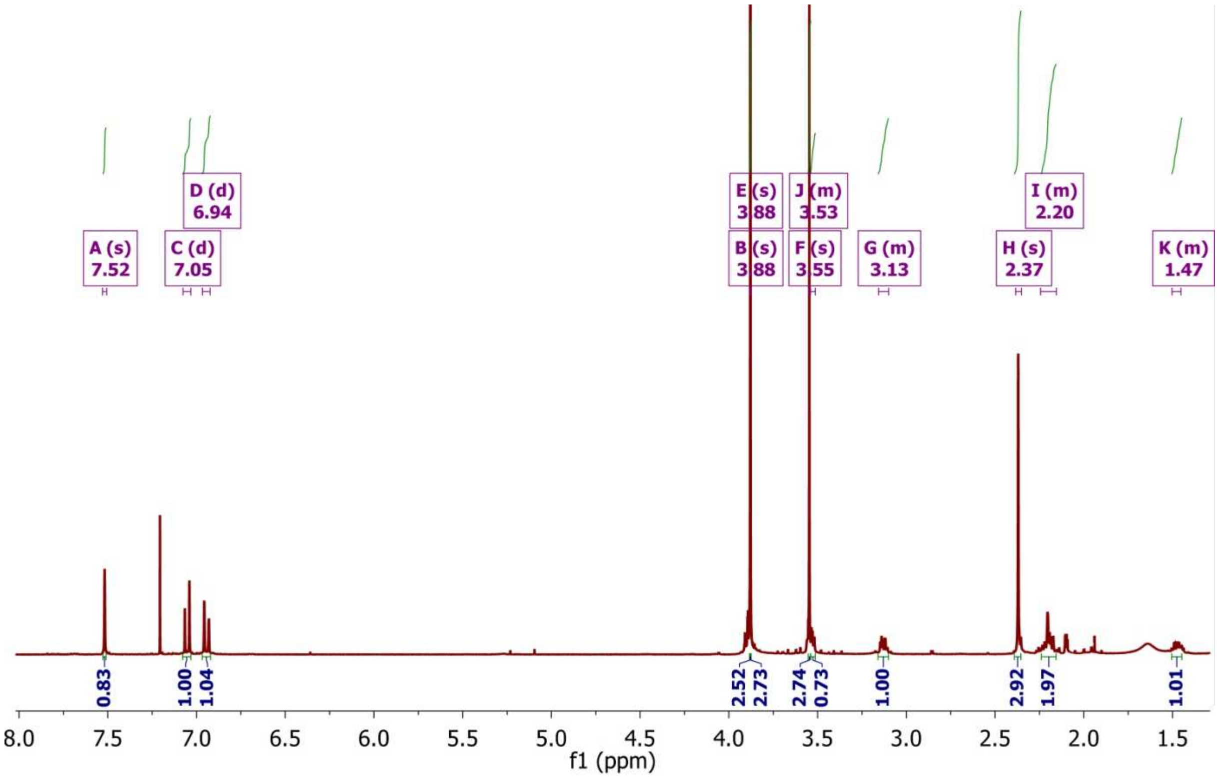

Figure S6. The ^{1}H NMR spectrum of **4** in $CDCl_3$.

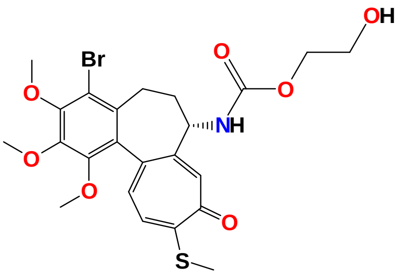

Chemical formula: $C_{23}H_{26}BrNO_7S$, MW = 539.1 g/mol

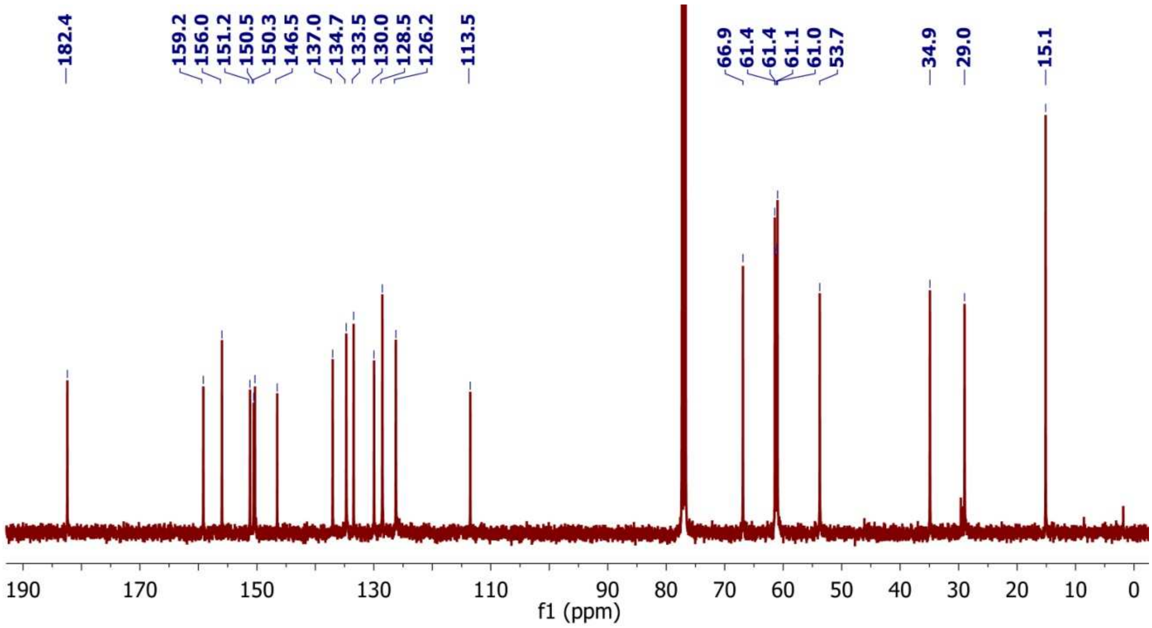

Figure S7. The ^{13}C NMR spectrum of **5** in $CDCl_3$.

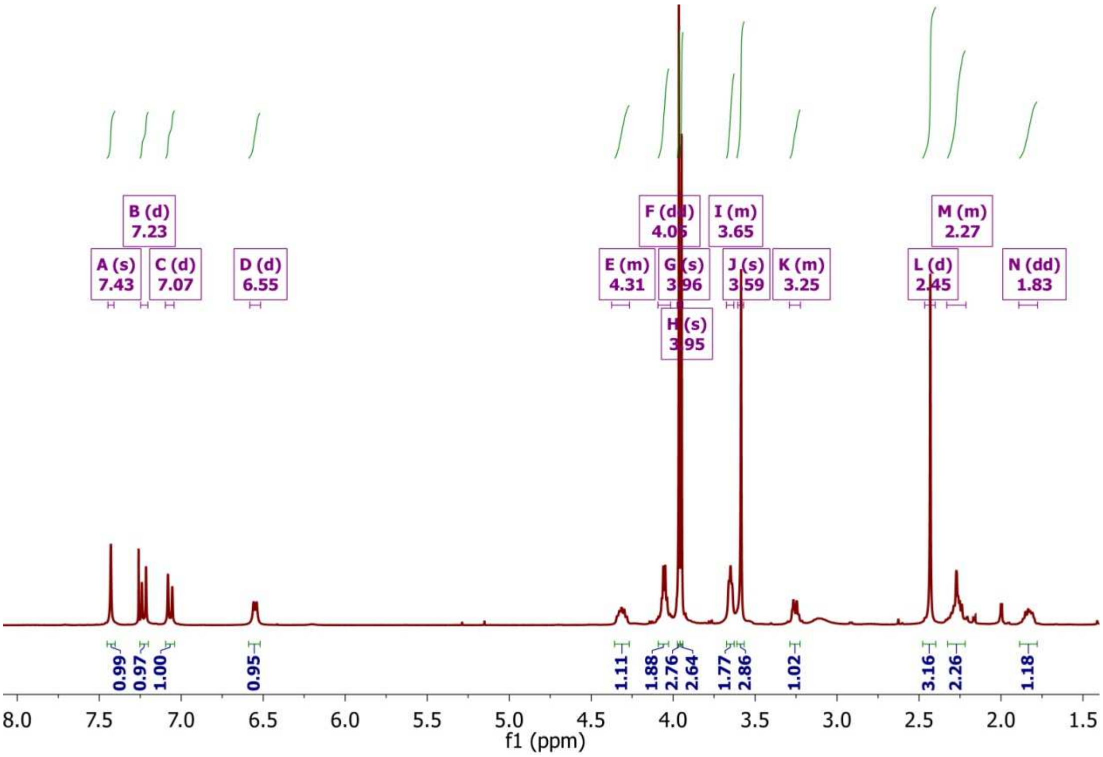

Figure S8. The ^{1}H NMR spectrum of **5** in $CDCl_3$.

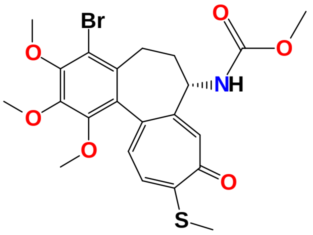

Chemical formula: $C_{22}H_{24}BrNO_6S$, MW = 509.1 g/mol

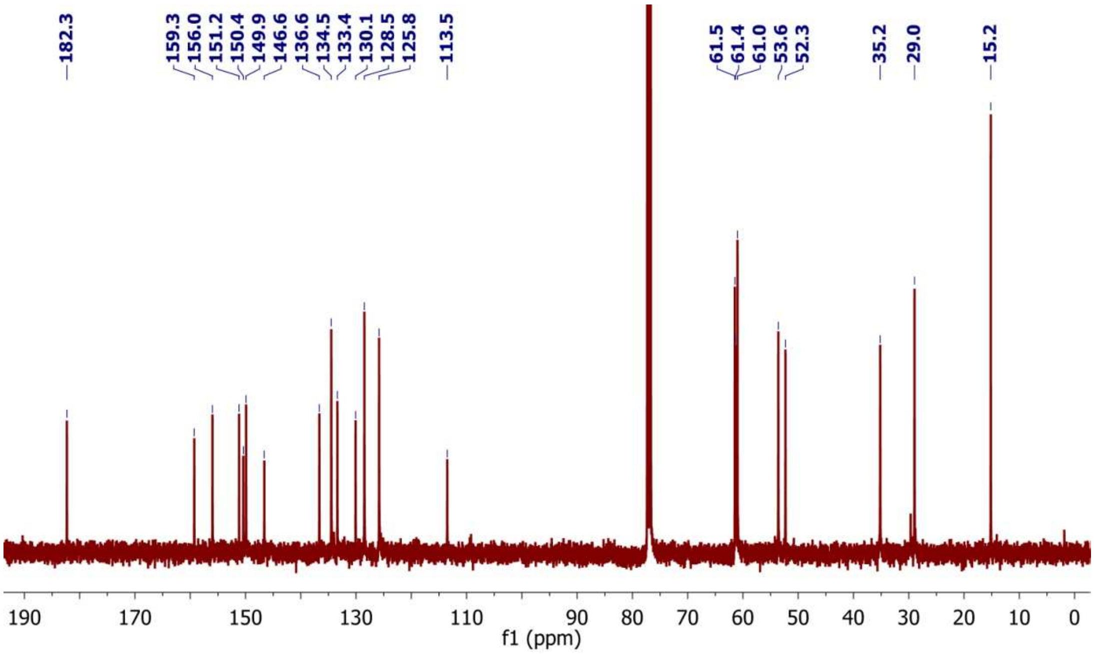

Figure S9. The ^{13}C NMR spectrum of **6** in $CDCl_3$.

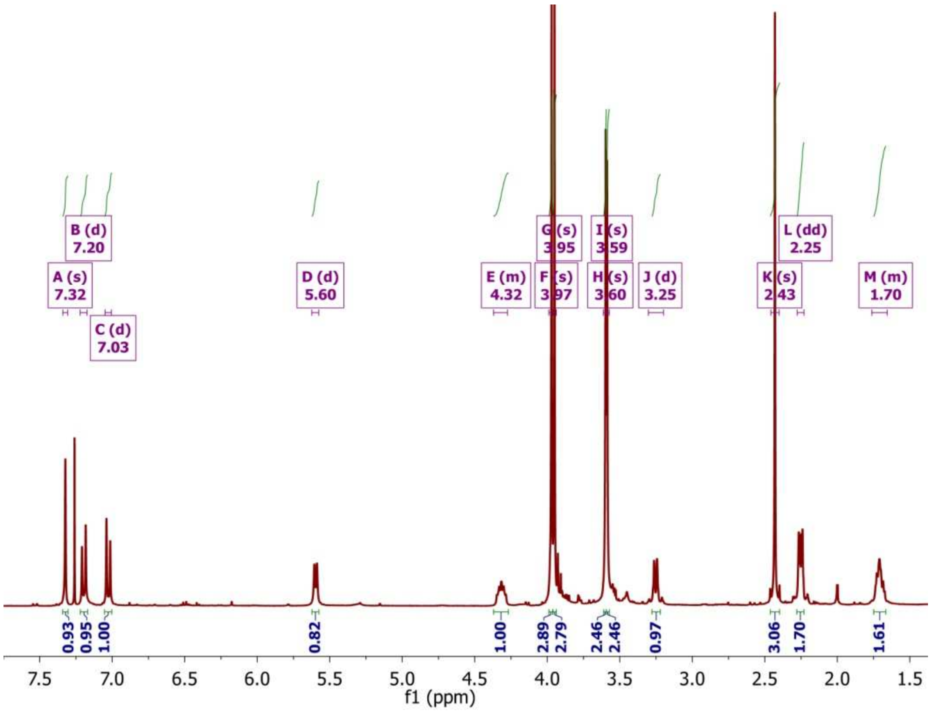

Figure S10. The ^{1}H NMR spectrum of **6** in $CDCl_3$.

cells

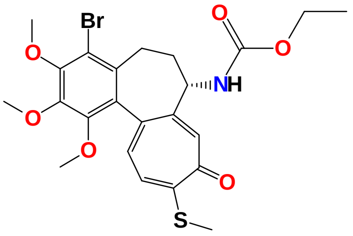

Chemical formula: $C_{23}H_{26}BrNO_6S$, MW = 524.4 g/mol

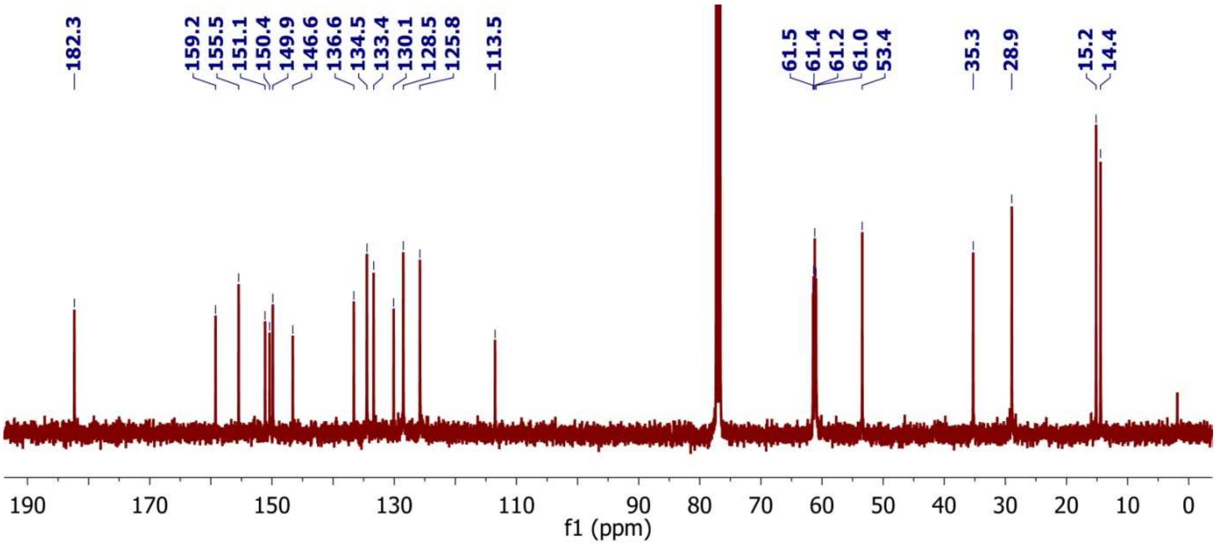

Figure S11. The ^{13}C NMR spectrum of **7** in $CDCl_3$.

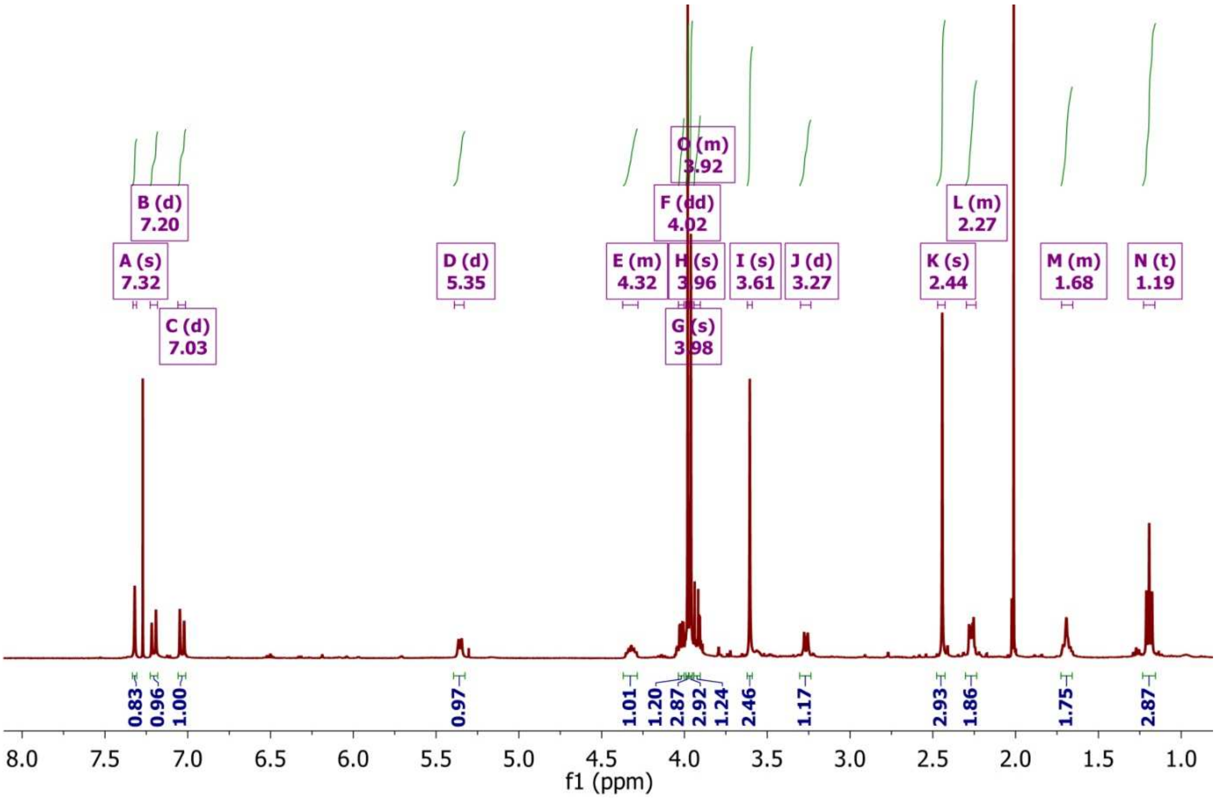

Figure S12. The ^{1}H NMR spectrum of **7** in $CDCl_3$.

cells

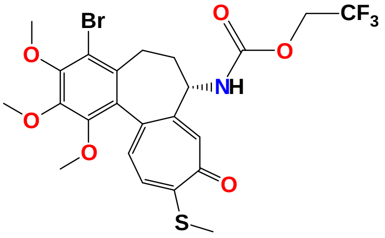

Chemical formula: $C_{23}H_{23}BrF_3NO_6S$, MW = 578.4 g/mol

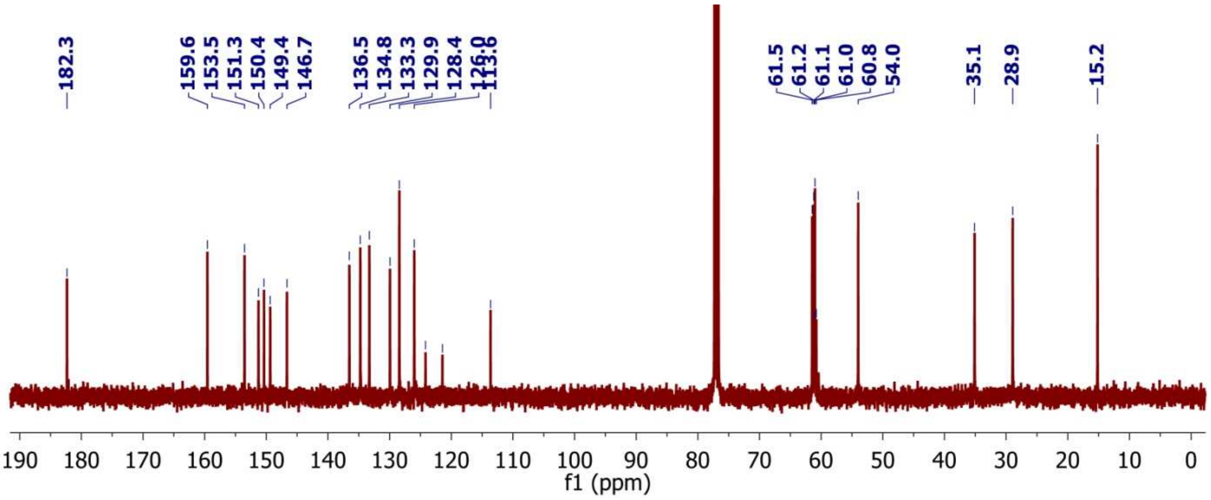

Figure S13. The ^{13}C NMR spectrum of **8** in $CDCl_3$.

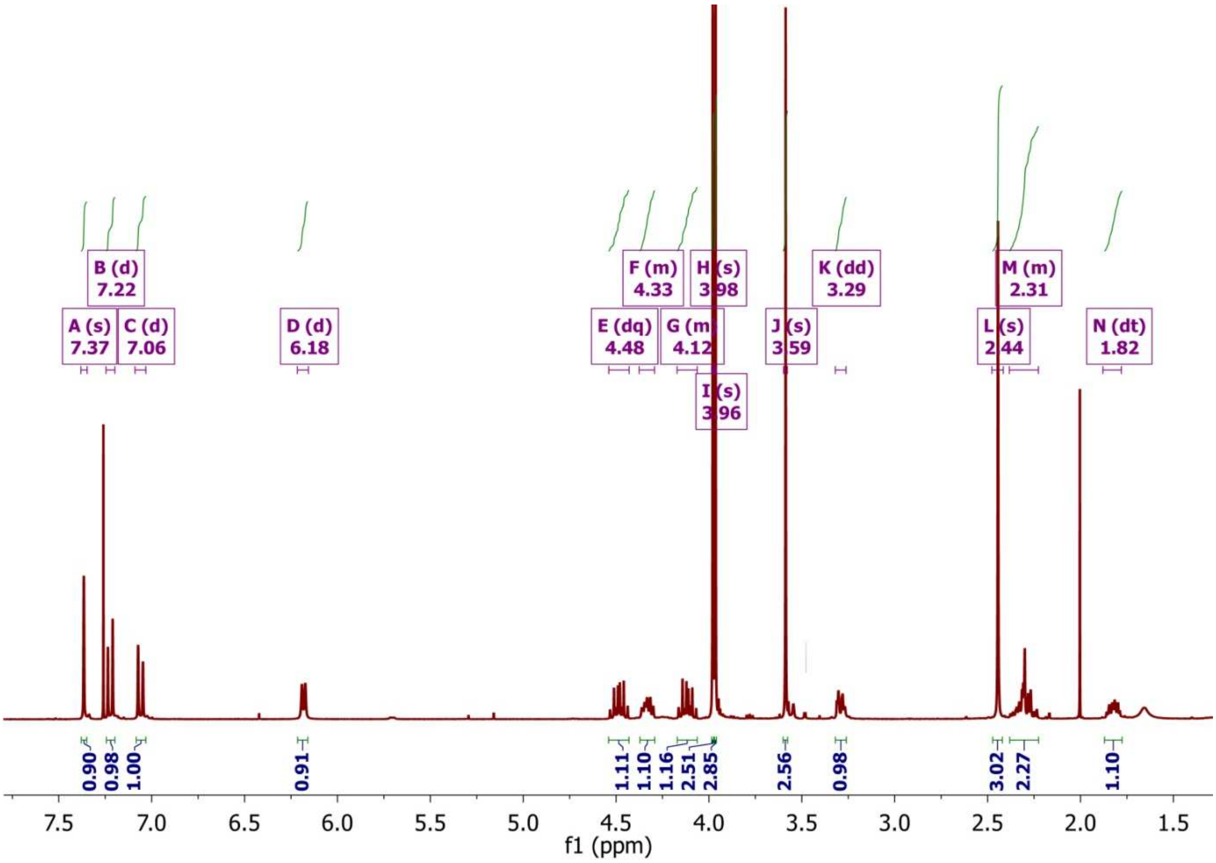

Figure S14. The ^{1}H NMR spectrum of **8** in $CDCl_3$.

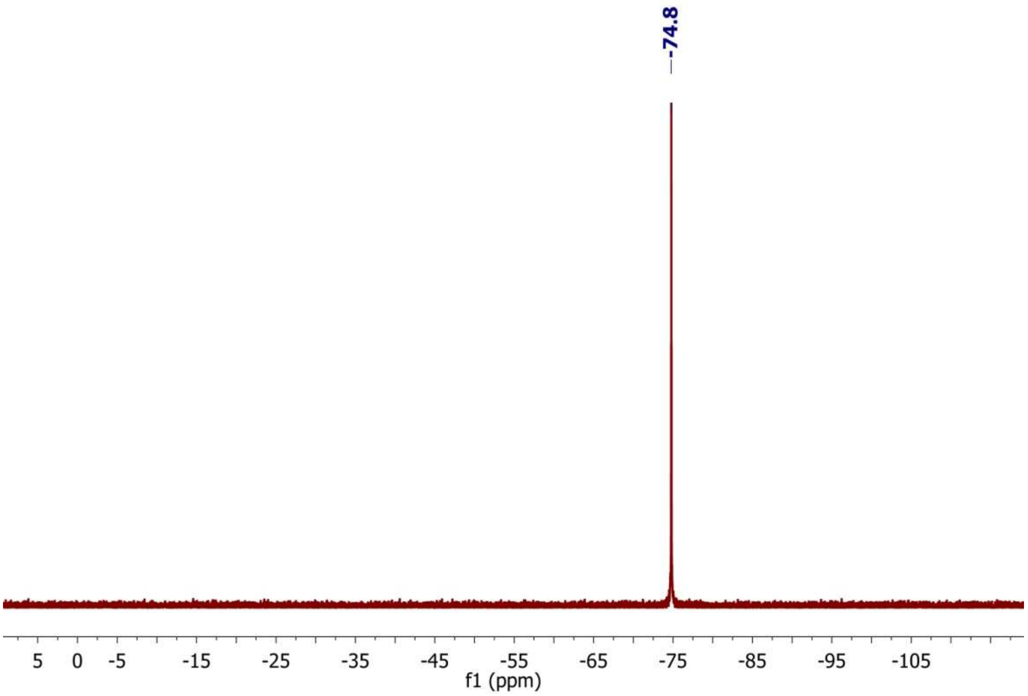

Figure S15. The ^{19}F NMR spectrum of **8** in $CDCl_3$.

cells

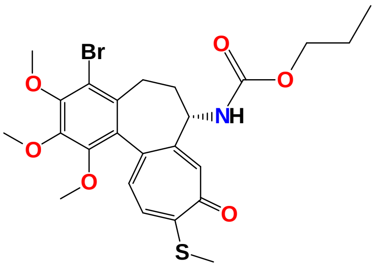

Chemical formula: $C_{24}H_{28}BrNO_6S$, MW = 538.5 g/mol

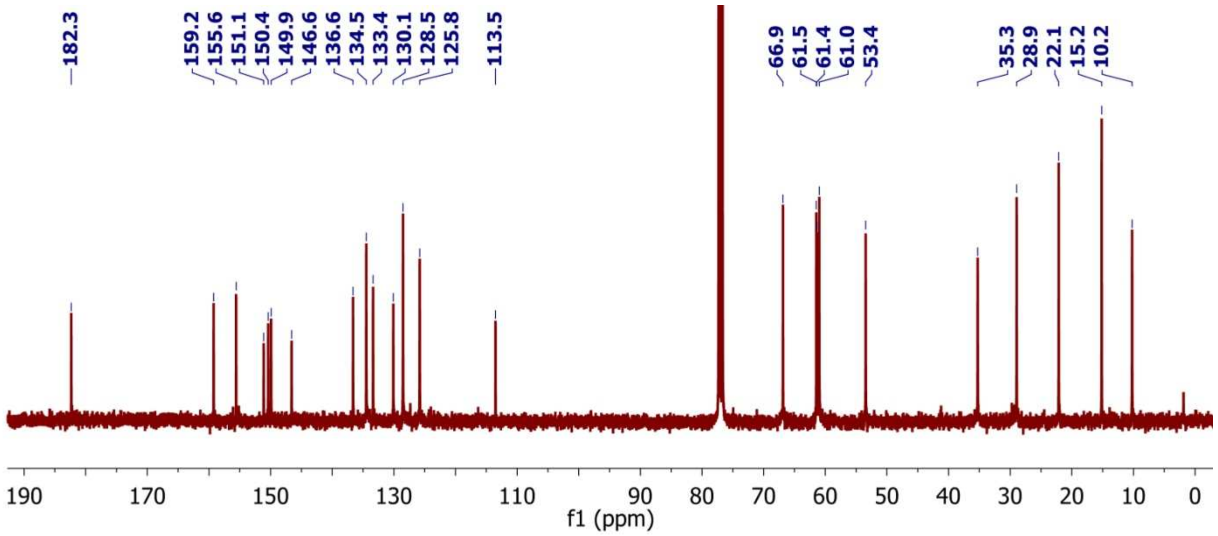

Figure S16. The ^{13}C NMR spectrum of **9** in $CDCl_3$.

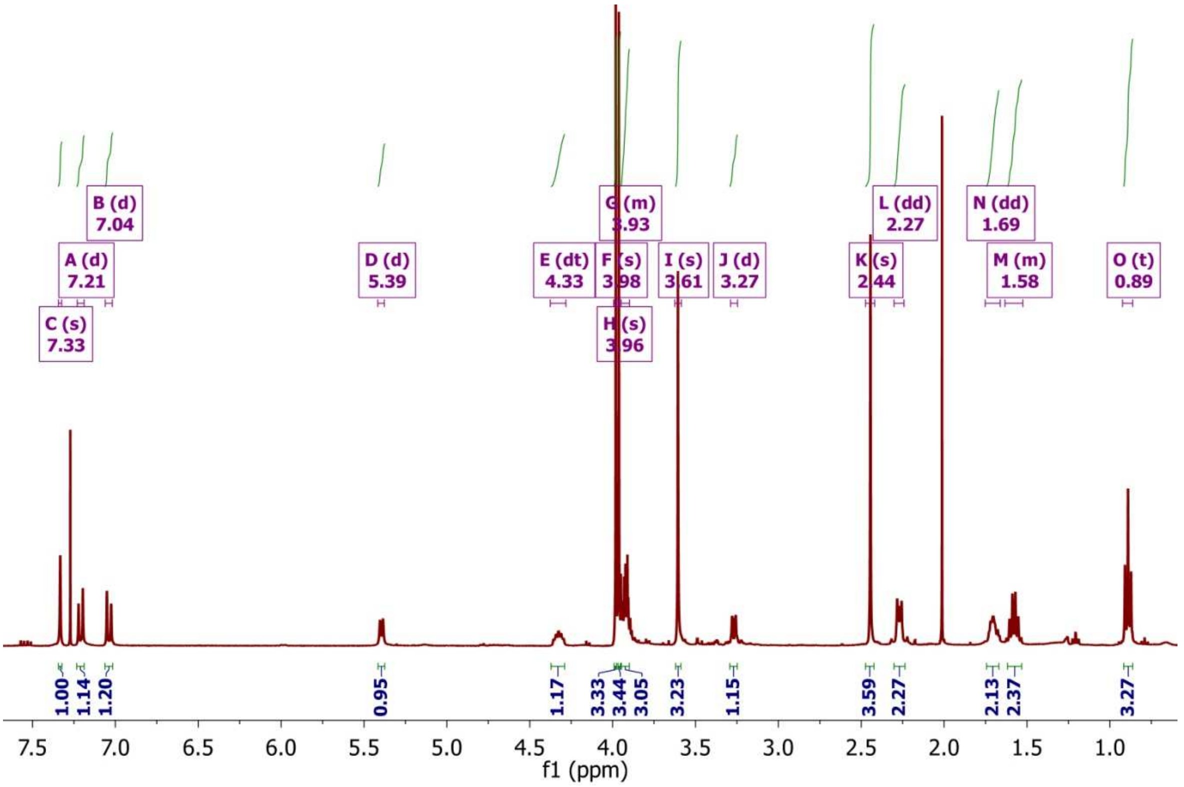

Figure S17. The ^{1}H NMR spectrum of **9** in $CDCl_3$.

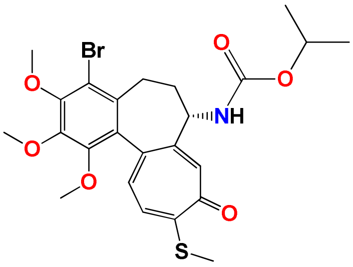

Chemical formula: $C_{24}H_{28}BrNO_6S$, MW = 538.5 g/mol

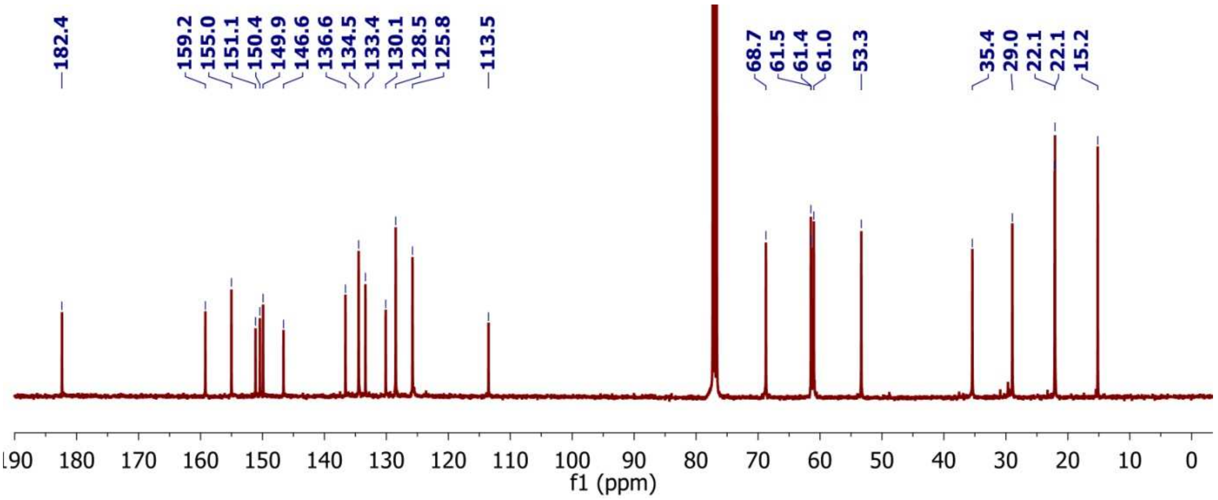

Figure S18. The ^{13}C NMR spectrum of **10** in $CDCl_3$.

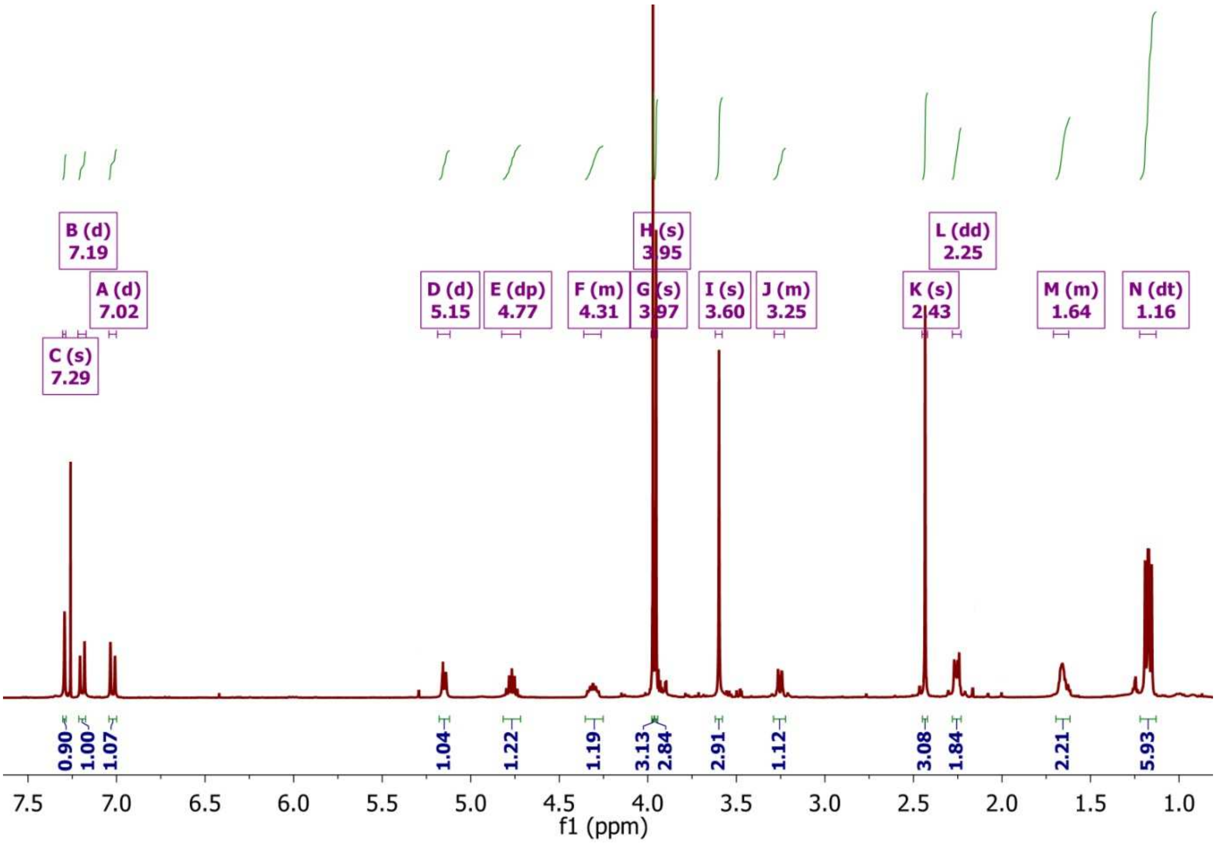

Figure S19. The ^{1}H NMR spectrum of **10** in $CDCl_3$.

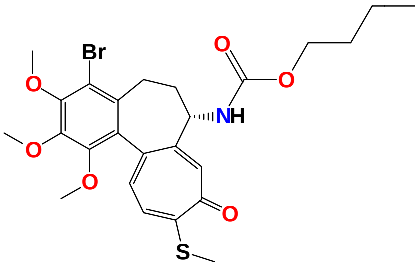

Chemical formula: $C_{25}H_{30}BrNO_6S$, MW = 551.1 g/mol

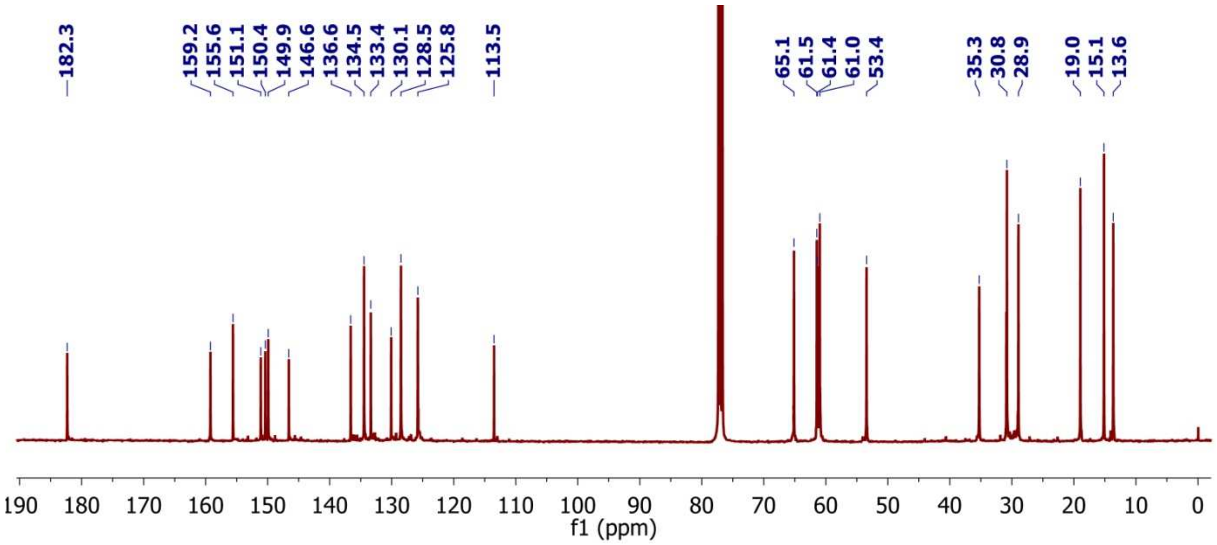

Figure S20. The ^{13}C NMR spectrum of **11** in $CDCl_3$.

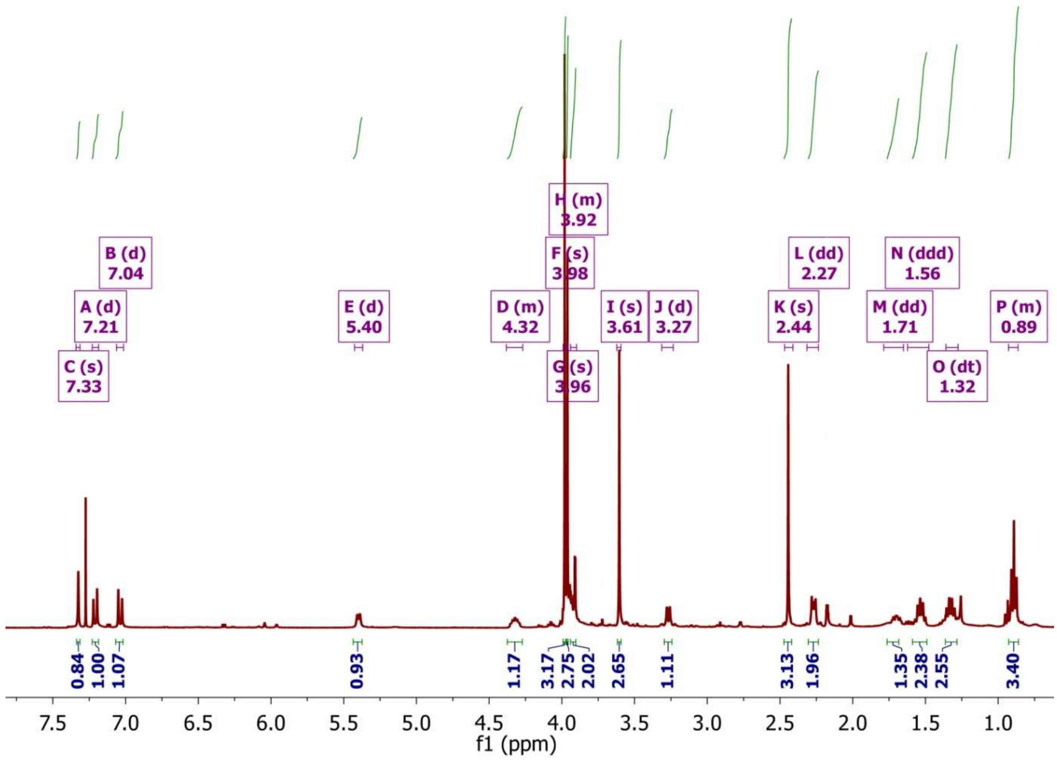

Figure S21. The ^{1}H NMR spectrum of **11** in $CDCl_3$.

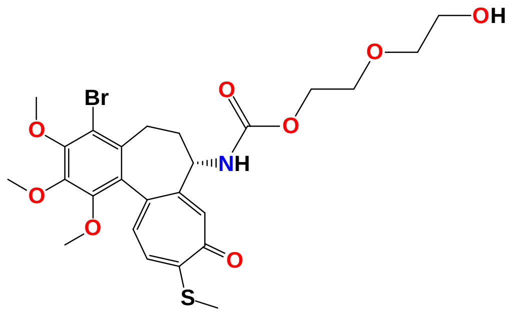

Chemical formula: $C_{25}H_{30}BrNO_8S$, MW = 584.5 g/mol

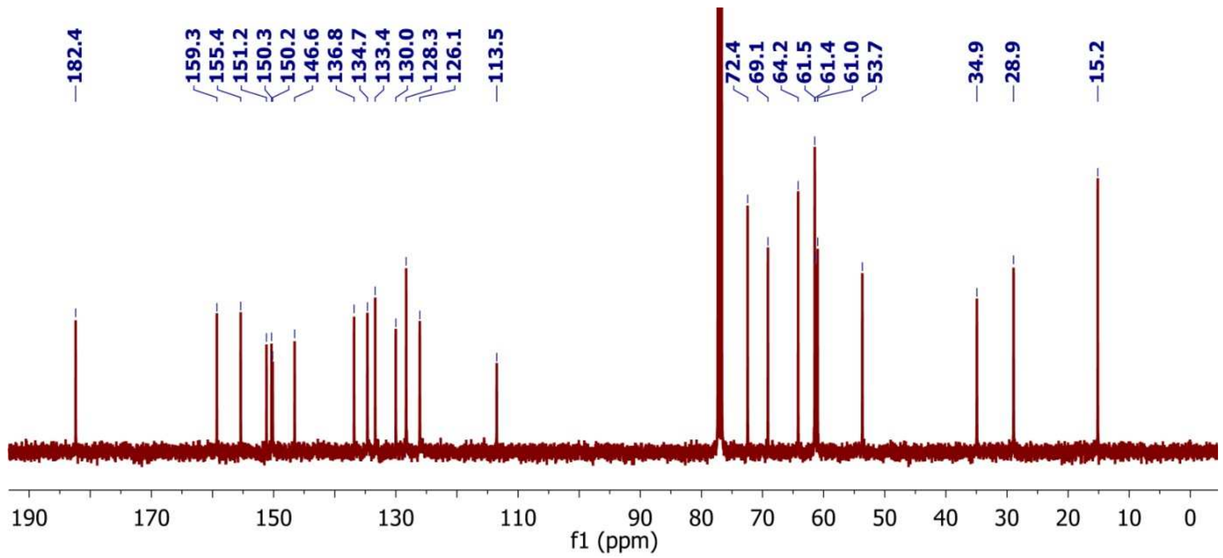

Figure S22. The ^{13}C NMR spectrum of **12** in $CDCl_3$.

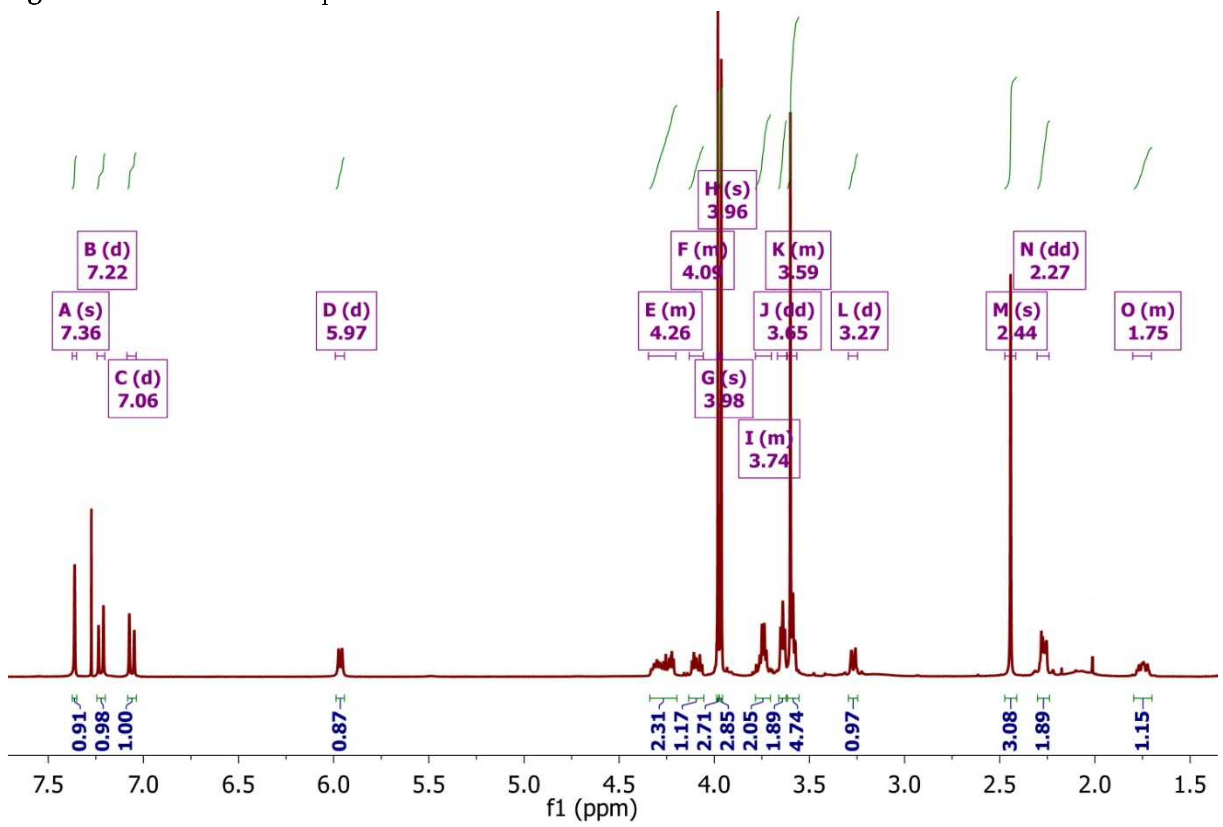

Figure S23. The ^{1}H NMR spectrum of **12** in $CDCl_3$.

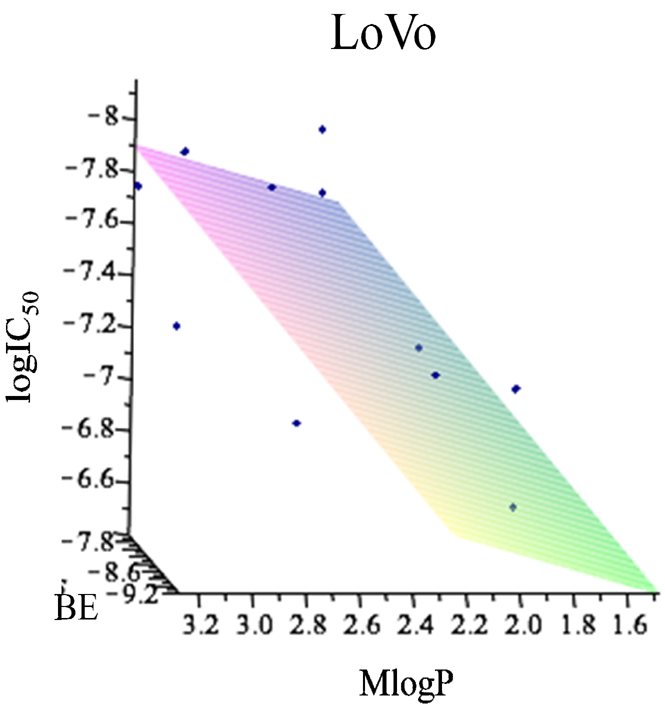

Figure S24. 3D plots of the linear regression results for the binding energies (BE) vs MlogP vs log IC_{50} [μM] values for LoVo cell line.

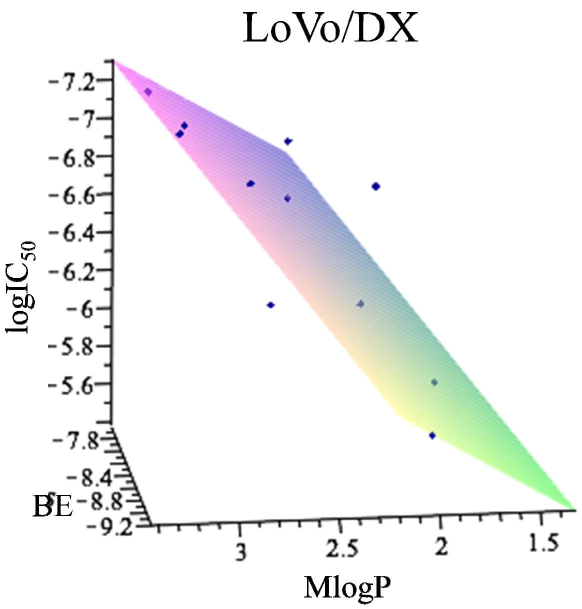

Figure S25. 3D plots of the linear regression results for the binding energies (BE) vs MlogP vs log IC_{50} [μM] values for LoVo/DX cell line.
